# Production of human papillomavirus type 16 virus-like particles in Physcomitrella photobioreactors

**DOI:** 10.1007/s00299-025-03602-x

**Published:** 2025-09-17

**Authors:** Paul Alexander Niederau, Maria Caroline Weilguny, Sarah Chamas, Caitlin Elizabeth Turney, Juliana Parsons, Marta Rodríguez-Franco, Sebastian N. W. Hoernstein, Eva L. Decker, Henrik Toft Simonsen, Ralf Reski

**Affiliations:** 1 Plant Biotechnology, Faculty of Biology, University of Freiburg, Schänzlestr. 1, 79104 Freiburg, Germany; 2 Department of Biotechnology and Biomedicine, Technical University of Denmark, 2800 Kongens Lyngby, Denmark; 3 Cell Biology, Faculty of Biology, University of Freiburg, Schänzlestr. 1, 79104 Freiburg, Germany; 4 Laboratoire de Biotechnologies Végétales appliquees aux Plantes Aromatiques et Médicinales, CNRS, UMR 5079, Université Jean Monnet, 42023 Saint-Etienne Cedex 2, France; 5 Signalling Research Centre BIOSS and CIBSS, University of Freiburg, Schänzlestr. 18, 79104 Freiburg, Germany; 6 Cluster of Excellence livMatS @ FIT – Freiburg Centre for Interactive Materials and Bioinspired Technologies, University of Freiburg, Georges-Köhler-Allee 105, 79110 Freiburg, Germany

**Keywords:** Bryophyte, Bioreactor, Molecular farming, Nanoparticle, Plant biotechnology, Vaccine candidate

## Abstract

**Key message:**

**First production of virus-like particles as a vaccine candidate in a non-vascular plant.**

**Abstract:**

Virus-like particles (VLPs) are self-assembling nanoparticles composed of viral structural proteins which mimic native virions but lack viral DNA and infectivity. VLPs are a resourceful class of biopharmaceuticals applied as subunit vaccines or as delivery vehicles for drugs and nucleic acids. Similar to viruses, VLPs are diverse in structure, composition, and assembly, requiring a tailored production platform aligned with the intended application. The moss plant Physcomitrella (*Physcomitrium patens*) is an emerging expression system offering humanized N-glycosylation, scalability, and adaptability to existing industry settings. Here, we used Physcomitrella to produce human papillomavirus (HPV) 16 VLPs. HPV VLPs are composed of the major structural protein L1 and are used as vaccines against HPV infections which are the main causal agent of cervical and other anogenital cancers. We characterized Physcomitrella chloroplast transit peptides, which we used for targeting of moss-produced L1 to chloroplasts, leading to higher recombinant protein yield compared to nuclear or cytoplasmic localization. We confirmed subcellular localization with confocal laser scanning microscopy and found L1 to accumulate within the chloroplast stroma. Production in 5-L photobioreactors yielded over 0.3 mg L1 per gram fresh weight. We established a purification protocol for moss-produced L1 using a combination of ammonium sulphate precipitation and cation exchange chromatography. Purified samples were subjected to a controlled dis- and reassembly, yielding fully assembled HPV-16 L1 VLPs. This is the first report of production, purification, and assembly of VLPs in a non-vascular plant.

**Supplementary Information:**

The online version contains supplementary material available at 10.1007/s00299-025-03602-x.

## Introduction

Virus-like particles (VLPs) are nanoparticles consisting of viral structural proteins (Marsian and Lomonossoff 2016; Nooraei et al. 2021). VLPs cannot replicate and infect cells, but resemble native viruses in size, shape, and surface structure, presenting highly repetitive immunogenic epitopes. As a result, VLPs have a wide range of applications as vaccines and nanocarriers and are of high interest in biopharmaceutical research and development.

There is currently a range of VLP-based vaccines licensed and available against infectious diseases such as hepatitis B, hepatitis E, human papillomavirus (HPV), malaria, and human Norwalk virus with many more vaccine candidates in clinical trials (Nooraei et al. 2021; Gupta et al. 2023). Viral capsid proteins stimulate both the cellular and the humoral immune response, leading to the generation of cytotoxic and memory T cells as well as the production of specific antibodies via B cells (Ross et al. 2009; Song et al. 2011; Serradell et al. 2019; Chi et al. 2024). VLP-based vaccines offer improved safety as compared to live-attenuated or inactivated vaccines, which carry the risk of reverting to a pathogenic form (Burns et al. 2014). At the same time, VLPs don’t suffer the limitations of DNA-based vaccines, including risk of genome integration or inappropriate delivery which ultimately lowers immunogenicity (Lu et al. 2024). VLPs are not only used for their intrinsic antigenic properties but serve as nanoparticles for drug delivery by loading their inner space with cargo such as nucleic acids (Lamprecht et al. 2016; Adams et al. 2023). Besides the inner space, VLPs can also be used to display antigens on the capsid’s surface. For the treatment of pancreatic cancers, disease-unrelated VLPs were coated with respective antigens and were demonstrated to inhibit cancer growth (Cubas et al. 2011; Zhang et al. 2013). Yet, empty VLPs of Cowpea Mosaic Virus, a rod-like shaped plant virus, were shown to reduce cancer growth, despite the absence of disease-specific antigens in the vaccine (Lizotte et al. 2015). The ability of VLPs to induce strong T cell-mediated immune responses is essential in VLP-based cancer vaccines (Nooraei et al. 2021). Overall, VLPs possess characteristics that make them highly versatile for applications in vaccine development, targeted drug delivery, gene therapy, and immunotherapy, offering significant potential in modern biomedicine.

VLPs are classified based on various structural characteristics, one of which is the presence or absence of a lipid envelope (Nooraei et al. 2021). In enveloped VLPs (eVLPs) the viral capsid proteins are imbedded within a lipid membrane acquired during particle assembly, typically from intracellular membranes in the secretory pathway or the outer plasma membrane. Additional structural characteristics include the number and types of capsid proteins, as well as their organization into single, double, or triple-layered architectures. In some instances, capsid proteins require proteolytic processing or N-glycosylation to enable budding of assembled particles from the host cell’s membrane and acquisition of the lipid envelope (Welsch et al. 2007; Zlotnick and Mukhopadhyay 2011; Kim et al. 2014). Furthermore, viral antigens displayed on the surface of the lipid membrane are often glycosylated, which significantly contributes to the immunogenicity of eVLP-based vaccines (Mortola and Roy 2004). One example is the major glycoprotein of Ebola virus for which the distinct glycosylation pattern is crucial for the formation of neutralizing antibodies (Peng et al. 2022). Other notable cases include hepatitis C virus and Zika virus—both of which currently lack a licensed vaccine—where the induction of neutralizing antibodies depends on viral glycoproteins (Torresi 2017; Hasan et al. 2018). Consequently, the choice of VLP, its structural complexity, and intended application play a crucial role in determining the appropriate expression system and guiding cell line development.

The moss Physcomitrella has emerged as an alternative for biopharmaceutical production, offering several advantages over traditional systems (Reski et al. 2015; Reski 2018; Decker and Reski 2020). Its haploid dominant phase and high rate of homologous recombination in somatic cells enable precise gene targeting (Strepp et al. 1998; Wiedemann et al. 2018; Rempfer et al. 2022). This feature facilitated the modification of the N-glycosylation pathway, eliminating plant-specific modifications, such as β1,2-xylosylation, α1,3-fucosylation, and Lewis A epitopes (Koprivova et al. 2004; Parsons et al. 2012; Decker et al. 2014). Moreover, enzymes necessary for sialic acid synthesis, activation, and linkage to protein N-glycans were introduced (Bohlender et al. 2020, 2022). These modifications have resulted in more homogeneous and human-like N-glycan structures on recombinant proteins. The potential of Physcomitrella as a biopharmaceutical production platform is demonstrated by several products in development. For example, moss-produced α-galactosidase A has successfully completed clinical phase Ib (Shen et al. 2016; Hennermann et al. 2019). Other moss-produced proteins include human factor H (FH) and synthetic FH-related multitarget regulators MFHR1 and MFHR13, which are potential treatments for complement-related disorders (Büttner-Mainik et al. 2011; Michelfelder et al. 2017, 2018; Top et al. 2019; Ruiz-Molina et al. 2022a) such as age-dependent macular degeneration AMD (Hector et al. 2025). Factor H produced in other systems faced difficulties in its application, such as *Pichia pastoris*-produced factor H exhibiting non-optimal glycosylation and reduced half-life (Schmidt et al. 2011; Kerr et al. 2021). Importantly, Physcomitrella cultivation in photobioreactors is tightly controlled (Hohe et al. 2002), and thus compliant with Good Manufacturing Practice (GMP) standards, ensuring high-quality production of recombinant proteins (Reski et al. 2018; Decker and Reski 2020). As opposed to transiently transfected *Nicotiana benthamiana* plants cultivated in green houses, cultivation of stable transgenic Physcomitrella cell lines in bioreactors is more in line with current production processes applied in industry, thereby facilitating adaption to industry settings (Benvenuto et al. 2023). In addition, cultivation of plant material in bioreactors allows greater scalability, which is a relevant aspect in transitioning to industrial scale production. The company Medicago, focusing on VLP production in transiently transfected *N. benthamiana*, announced challenges in transitioning with their platform to industrial scale production as a reason for ceasing further operations in 2023 (Mitsubishi Chemical Group Corporation 2023). In contrast, the company Eleva upscaled protein production in Physcomitrella from 200 L single-use bioreactors (Niederkrüger et al. 2019) to 1000 L photobioreactors (Eleva 2025). In summary, Physcomitrella is an ideal production host for complex biopharmaceuticals, including viral antigens or glycosylated proteins, with great prospects regarding scalability and transition to industrial production (Rosales-Mendoza et al. 2014). The first moss-produced vaccine candidate, an ENV-derived multi-epitope HIV chimeric protein, was immunogenic in mice (Orellana-Escobedo et al. 2015). However, until now, there was no record of VLP-production in Physcomitrella, or any other non-vascular plant.

VLPs of the human papillomavirus (HPV) are of great medicinal relevance due to their use as vaccines to prevent HPV infection. HPV infections are a major source of cancer, causing approximately 690,000 cases annually (zur Hausen et al. 1981; zur Hausen 2002; de Martel et al. 2020). The most prevalent type is cervical cancer, accounting for about 80% of HPV-attributable cancers. Other HPV-related cancers include anogenital and oropharyngeal cancers (Wu et al. 2024). HPV belongs to the *Papillomaviridae*, comprising nearly 200 different types, which are commonly transmitted sexually (Ljubojevic and Skerlev 2014). The majority of HPV-attributable cancers are linked to two types in particular, namely HPV-16 and HPV-18 (de Martel et al. 2020; Wu et al. 2024). HPV is a non-enveloped double stranded DNA virus with a genome size of 8000 bp, encoding six early regulatory proteins (E1, E2, E4–E7) and two late structural proteins (L1, L2) (Roden and Stern 2018). L1 proteins multimerize into pentamers, the capsomers, and L1 capsomers and L2 monomers are imported into the nucleus via nuclear localization signals (NLS), where HPV virions assemble (Cerqueira and Schiller 2017). The final HPV-16 virion is composed of 360 L1 monomers, organized in 72 pentamers, and 72 L2 monomers and is reinforced by inter-pentameric disulfide bonds (Baker et al. 1991; Buck et al. 2005). While the role of L2 in virion assembly differs from type to type, L1 can self-assemble into VLPs in the absence of L2 (Kirnbauer et al. 1992). HPV L1 VLPs are mono-layered and do not contain a lipid envelope. Because the viral life cycle depends on differentiated epithelial cells, it is difficult to obtain large quantities of virions. Therefore, current HPV vaccines are based on L1 VLPs. Although VLP-based HPV vaccines are currently produced in *Escherichia coli*, *Saccharomyces cerevisiae*, and baculovirus-infected insect cells (World Health Organization 2024), plant-based expression systems are gaining attention in this respect (Scotti and Rybicki 2013; Rybicki 2020).

While first attempts to produce L1-based VLPs in plants had low yields (Biemelt et al. 2003; Varsani et al. 2003), considerable improvements have been achieved, e.g. by codon optimization and subcellular targeting strategies in transiently transformed tobacco (Maclean et al. 2007). A human codon-optimized version (62% GC content) resulted in highest yields as compared to *N. benthamiana* optimized (49% GC) and the native sequence (37% GC), presumably not due to the GC content but due to inhibitory 5’ sequences, which act on transcription and mRNA processing (Maclean et al. 2007; Hitzeroth et al. 2018). Further, these authors found chloroplast-targeted L1 yield highest at 0.5 mg/g fresh weight (FW) followed by cytoplasmic L1 (without the C-terminal 22 aa NLS), whereas L1 targeted to the ER was not detectable. Similarly, Zahin et al. (2016) reported 0.25 mg L1/g FW via chloroplast targeting while leaving the NLS intact. Although *N. benthamiana* remains a prominent option, other plants are increasingly explored for the efficient production of biopharmaceuticals.

Here, we analysed the expression of HPV-16 L1 in Physcomitrella. We characterized five Physcomitrella chloroplast transit peptides and investigated the effect of subcellular L1 localization on recombinant protein yields. We further established L1 production in photobioreactors and subsequent purification of the product. Finally, the assembly of moss-produced HPV-16 L1 to VLPs was demonstrated with transmission electron microscopy. Our goal was to obtain proof-of-concept to produce a simple but medicinally relevant VLP before progressing towards more complex targets such as eVLPs consisting of multiple proteins and layers.

## Results

### Adaptation of L1 coding sequence for Physcomitrella-optimized expression

Sometimes, Physcomitrella splices RNAs transcribed from stably integrated heterologous cDNAs, resulting in aberrant protein isoforms and low product yields, a process referred to as heterosplicing (Top et al. 2021). To prevent such unwanted effects that would compromise the final product, we optimized the coding sequence (CDS) of HPV-16 L1 for expression in Physcomitrella according to established protocols (Top et al. 2021). The goal was to adapt the CDS regarding codon usage and preventing potential degradation through microRNAs and splicing. A human codon-optimized version (hL1, Supplementary Figure S2) was used as a basis for optimization since it had led to higher yields than plant-optimized versions or the native viral sequence in *Nicotiana benthamiana* (Maclean et al. 2007).

For codon optimization, physCO (Top et al. 2021) was used, which changed nine out of 505 codons and slightly increased the GC content from 62.25 to 62.85% (Supplementary Figure S2). Next, the CDS was scanned for the motif AGGT, which is the most common motif at exon–intron borders for alternative splicing in Physcomitrella (Top et al. 2021). Transformation of a heterologous cDNA containing the AGGT motif led to alternative splicing in Physcomitrella, a process referred to as heterosplicing (Top et al. 2021). Scanning the physCO-optimized hL1 sequence yielded 5 AGGT-motifs which were replaced by synonymous codons (Supplementary Figure S2). MicroRNAs bind complementary sequences in the mRNA and thereby cause inhibition of translation or degradation of mRNA (Jones-Rhoades et al. 2006; Khraiwesh et al. 2010). One microRNA binding site was predicted within the CDS using psRNATarget (Dai and Zhao 2011; Dai et al. 2018). Therefore, we changed three bases within the predicted binding region using synonymous codon substitutions. The final Physcomitrella-optimized CDS was named pL1 (Supplementary Figure S2) and differs from hL1 by 17 out of 1515 nucleotides without changing the amino acid (aa) sequence.

### Characterization of chloroplast transit peptides

Different constructs were designed to target L1 to specific cellular compartments, including chloroplasts. Fusing chloroplast transit peptides (CTPs) to the L1 CDS led to increased protein yields in tobacco as compared to constructs lacking CTPs (Maclean et al. 2007; Zahin et al. 2016). To assess whether this is also true for L1 production in Physcomitrella, a selection of Physcomitrella-derived CTPs was characterized.

Initially, the CTP of FtsZ1-1 (Gremillon et al. 2007) was considered. However, cleavage site analysis via TargetP-2.0 (Armenteros et al. 2019) predicted 37 aa of this CTP to remain at the N-terminus of L1 after chloroplast import. This CTP was rejected because the additional aa could change L1 properties and hamper VLP assembly. A list of alternative CTPs was derived from the Physcomitrella N-terminome (Hoernstein et al. 2024) (Supplementary Table S2). Here, experimentally detected protein N-termini were compared with TargetP-2.0 predictions for cleavable N-terminal targeting sequences. Five CTPs were selected where an exact match between prediction and experimentally validated N-terminus was observed across different experiments (Hoernstein al. 2024), namely CTPc5, CTPc10.1, CTPc10.2, CTPc21, CTPc22 (Table 1). Their sequences were fused in silico to the N-terminus of L1 and analyzed with TargetP-2.0 to validate the correct cleavage of the fusion.

**Table 1:** In-silico analysis of Physcomitrella-derived chloroplast transit peptides

Name of CTP	Originating from protein model	Predicted cleavage of CTP-L1	Length (aa)	Likelihood of GFP chloroplast localization	Likelihood of L1 chloroplast localization
FtsZ1-1	Pp3c22_4940V3.1	37 aa remain on N-terminus	84	0.950	0.920
CTPc5	Pp3c5_1370V3.1	–	74	0.987	0.943
CTPc10.1	Pp3c10_10490V3.1	–	63	0.456	0.229
CTPc10.2	Pp3c10_2940V3.1	–	70	0.908	0.845
CTPc21	Pp3c21_9980V3.1	–	43	0.956	0.891
CTPc22	Pp3c22_5470V3.1	–	62	0.939	0.739
CTPc22noMet	Pp3c22_5470V3.1	–	61	0.913	0.727

CTPs were selected based on mass spectrometry datasets (Hoernstein et al. 2024). In-silico predictions of likelihood of chloroplast localization target sequence cleavage were performed with TargetP-2.0.

Our in-silico analysis indicated CTPc22 to end with a methionine, which could introduce an alternative translation initiation site, resulting in translation of L1 without the CTP (Table 1). Therefore, an alternative version lacking the C-terminal methionine (CTPc22noMet) was included. The likelihoods for chloroplast localization were above 70% for all candidates except CTPc10.1 (Table 1), which was rejected.

The remaining five CTPs (CTPc5, CTPc10.2, CTPc21, CTPc22, CTPc22noMet) were fused to the CDS of eGFP (Supplementary Figure S1) and expressed in Physcomitrella protoplasts. Analysis via fluorescence microscopy confirmed localization of eGFP signals in chloroplasts for CTPc5, CTPc10.2, CTPc22, and CTPc22noMet, but not for CTPc21 (Supplementary Figure S3). Of the four remaining CTPs, CTPc5 had the highest likelihood for plastid localization (Table 1) and was examined via confocal microscopy (Supplementary Figure S3). Finally, we selected CTPc5 for the chloroplast-targeting of L1.

### Targeting of L1 to nuclei and chloroplasts

To evaluate the impact of L1 localization on product yield, we generated three variants (pL1, pL1∆22, CTP-pL1). pL1 contains the full-length pL1 CDS, which includes the native HPV-16 NLS (Cerqueira and Schiller 2017). For pL1Δ22 the NLS was removed. In CTP-pL1 the Physcomitrella CTPc5 was fused to the 5’ of the pL1 CDS, including the NLS.

After generation of these CDS variants, we aimed to evaluate L1 targeting to subcellular compartments. The CDS of pL1, pL1Δ22 and CTP-pL1 were fused to the 5′ end of Citrine CDS (Supplementary Figure S1). Physcomitrella protoplasts were transfected with the plasmids and Citrine localization was analysed using fluorescence microscopy. A vector encoding Citrine without any L1 variant fusion served as a control.

**Fig. 1:**
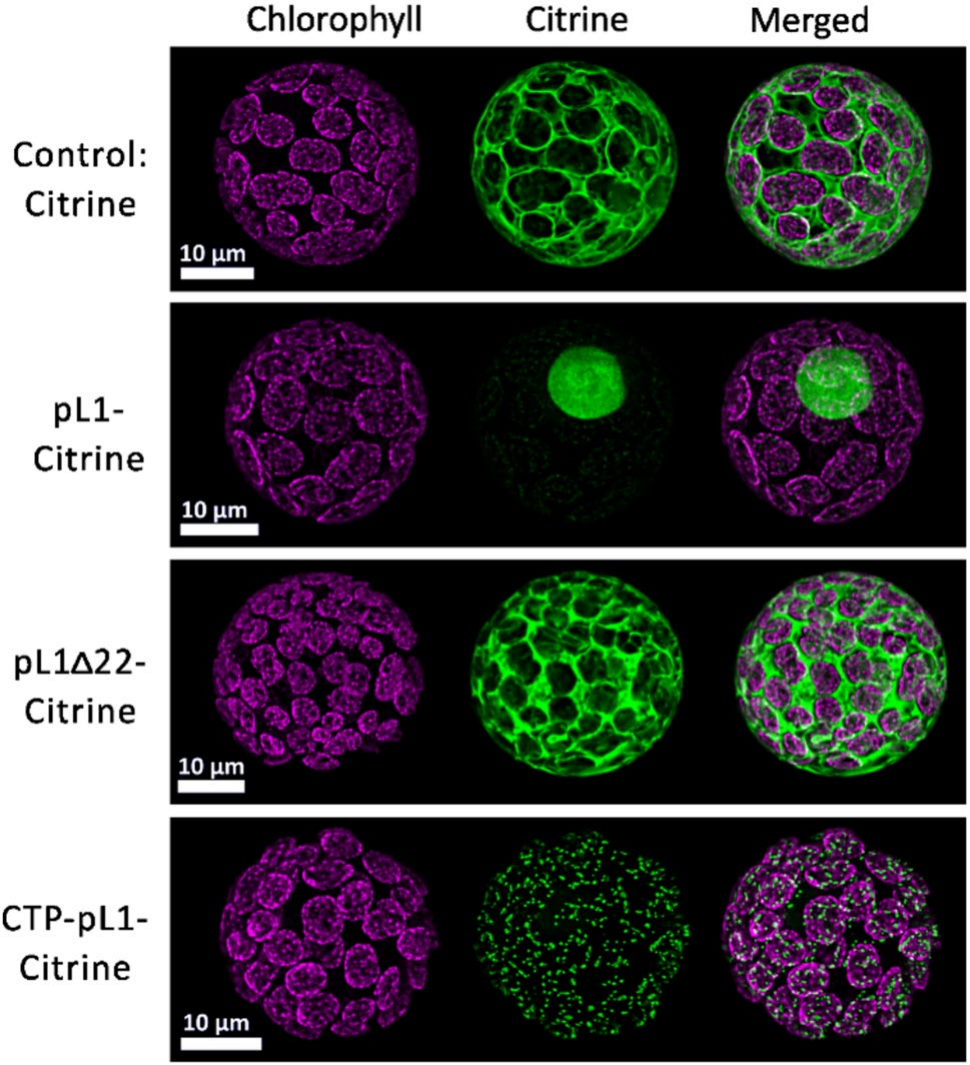
Subcellular localization of L1 variants. Confocal microscopy images of Physcomitrella protoplasts transformed with constructs encoding (CTP-)pL1(Δ22)-Citrine fusion proteins or Citrine alone. For each sample, chlorophyll autofluorescence in magenta is seen on the left, Citrine signals in the middle and the merged signals on the right. In the control and pL1Δ22-Citrine, the Citrine signals do not enter the chloroplasts and are mainly in the cytoplasm. For pL1-Citrine, the Citrine signals are in the nucleus. In the CTP-pL1-Citrine sample, the Citrine signals form small spots of high intensity in the chloroplasts.

Cells transformed with the control vector exhibited predominantly cytoplasmic Citrine signals, whereas diffuse signals also occurred in nuclei (Fig. 1). For pL1-Citrine, the signal localized to nuclei, demonstrating that the HPV-16 NLS is functional in Physcomitrella. This is further supported by the fact that removal of the NLS in pL1Δ22-Citrine results in cytoplasmic localization of the signal. For CTP-pL1-Citrine, the signal is predominantly in chloroplasts, but is not evenly distributed, as seen for CTP-eGFP fusions lacking L1 (Supplementary Figure S3). Instead, the CTP-pL1-Citrine fusion concentrates in several spots of high fluorescence intensity and less than 1 µm in diameter. It is unclear whether this difference is due to distinct features of GFP and Citrine or due to the presence of L1 and potential formation of L1 multimeric structures. Overall, we confirmed successful L1 targeting to the intended compartments.

### Characterization of L1 production

After validating the subcellular localization, pL1, pL1∆22 and CTP-pL1 expression constructs were cloned (Supplementary Figure S1). The linearized constructs were transferred into Physcomitrella protoplasts and obtained plants were screened for productivity by western blot and ELISA.

**Fig. 2:**
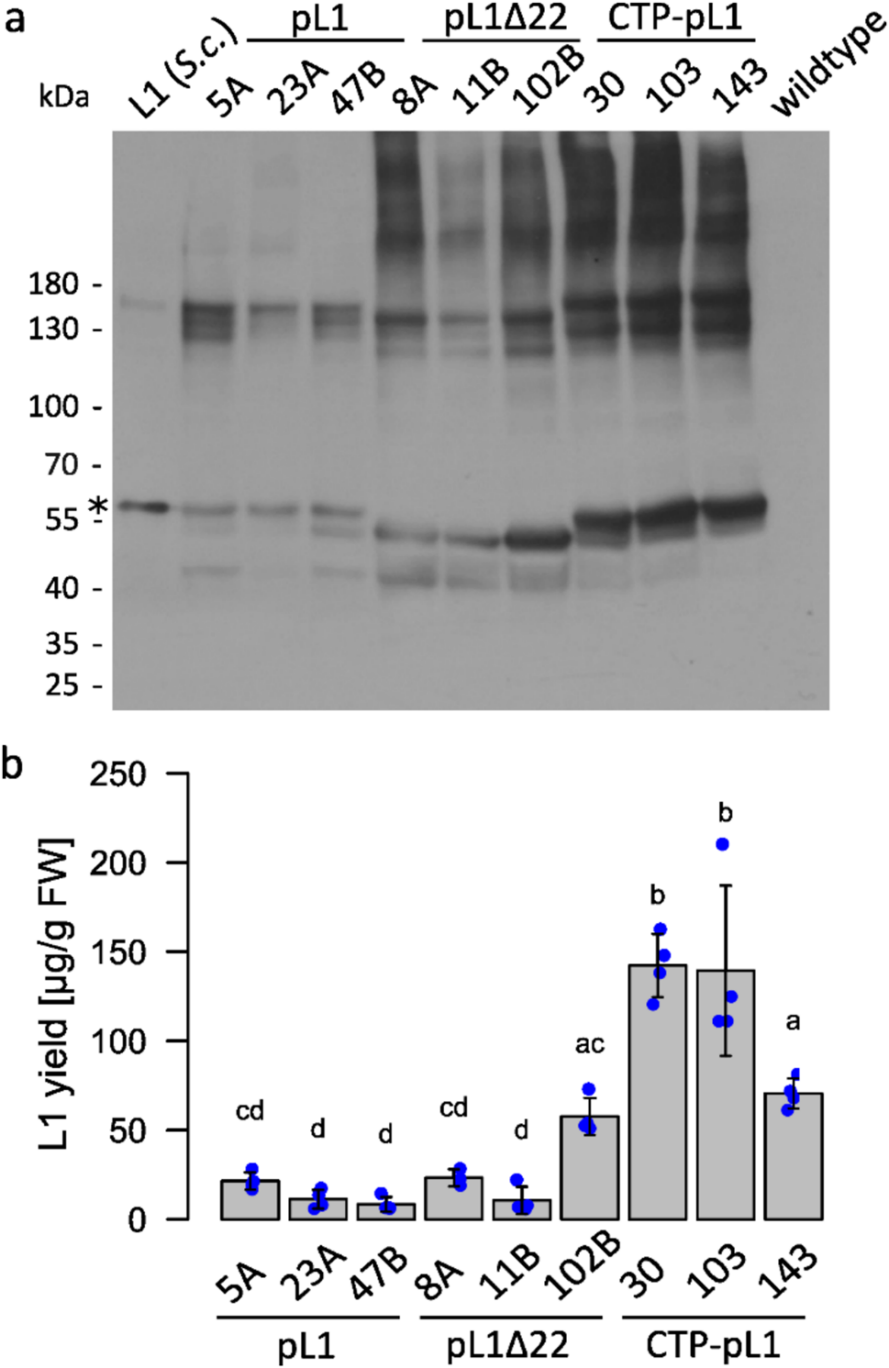
Analysis of HPV-16 L1 production via western blot and ELISA. **a** For each of the constructs pL1, pL1Δ22, and CTP-pL1 three lines were tested via western blot for expression of HPV-16 L1. 50 ng *S. cerevisiae*-produced L1 served as a positive control and Physcomitrella wildtype as a negative control. The proteins for the western blot were separated on a 7.5% TGS polyacrylamide gel under reducing conditions before being transferred to a membrane and developed using CamVir1 antibody (1:5000). The asterisk marks the band referring to full length HPV-16 L1. **b** Production levels of L1 were quantified via ELISA. Wildtype background signal was subtracted before analysis via one-way ANOVA and post hoc multiple comparisons test (TukeyHSD). Means and standard deviations are calculated based on n ≥ 3 technical replicates from two dilutions and are given per biomass fresh weight (FW). Statistical significance (α = 0.05) is indicated by Compact Letter Display.

Western blot analysis revealed the L1 monomer of 56 kDa (pL1, CTP-pL1) and 54 kDa (pL1Δ22) (Fig. 2). In addition, pL1 and CTP-pL1 lines exhibited a band at approximately 54 kDa. All lines showed another band at around 45 kDa, even though the signal was relatively weak in CTP lines and more prominent in pL1Δ22 lines. Smaller protein isoforms can originate from degradation or rearrangement of DNA or RNA, resulting in truncated transcripts and, subsequently, truncated protein isoforms (Murén et al. 2009; Top et al. 2021). The latter possibilities were ruled out by amplifying the L1 CDS via PCR from cDNA, which yielded amplicons of the expected sizes, corresponding to the western blot bands of 56 kDa (pL1, CTP-pL1) and 54 kDa (pL1Δ22), but not additional amplicons (Supplementary Figure S4). Also, two bands are visible at 130–180 kDa, of which the highest band at 180 kDa is also present in the yeast-produced L1 control. This pattern of two close-by bands repeats another two times at higher molecular weight outside of the marker range (Fig. 2). Addition of reducing agents DTT or ascorbic acid to the extraction buffer markedly reduces the western blot signal intensity above 180 kDa (Supplementary Figure S5). We hypothesize that in the absence of reducing agents, reactive oxygen species (ROS) in the plant extract cross-link L1 with itself and/or host proteins. Irrespective of the presence or absence of reducing agents during extraction and purification, two prominent bands in the range of 130 and 180 kDa are present in all western blots (Figs. 2, 4, Supplementary Figure S7). Western blot and ELISA analysis indicate differences in L1 expression levels between the constructs, showing higher yields in CTP-pL1 lines as compared to pL1 or pL1Δ22 lines. In shaking flasks, the best producer is CTP-pL1 30 with 142 µg L1/g FW (Fig. 2).

Besides the specific product yield, i.e. the amount of recombinant protein per g FW biomass, the accumulation of biomass is important in biomanufacturing as high specific product yields can be trivial if biomass accumulation is slow. To study if recombinant protein amount or subcellular localization of L1 hampers plant growth, we analysed the time course of biomass accumulation of the nine lines in shaking flasks for 4 weeks.

In our conditions, exponential growth could not be measured. This might be due to the culture conditions, to the rather long intervals between sampling, and/or to the intrinsic growth characteristics of Physcomitrella protonema, which grows by apical cell division (Reski 1998). Therefore, biomass density was fitted using linear regression analysis model (Supplementary Figure S6), using the calculated slope to approximate biomass accumulation rates. Comparison of the slopes between the lines pL1, pL1Δ22 and CTP-pL1 showed pL1 lines accumulated biomass faster than pL1Δ22 (p = < 0.0001) and CTP-pL1 (p = 0.0001) lines. If this difference is due to the distinct L1 localization or to lower L1 production in pL1 lines (Fig. 2) is unclear. Line pL1 47B showed the highest slope of 255 mg dry weight (DW)/L/week, as compared to line CTP-pL1 30 with a slope of 211 mg DW/L/week. However, this difference is negligible considering the yield of pL1 47B is 8 µg L1/g FW as to 142 µg L1/g FW for CTP-pL1 30 (Fig. 2). Consequently, line CTP-pL1 30 was chosen for VLP production.

### Upscaled L1 production

For production of VLPs, the cultivation of line CTP-pL1 30 was upscaled to 5 L-photobioreactors. The goal here was to identify the best time point for harvesting material used for subsequent L1 purification.

**Fig. 3:**
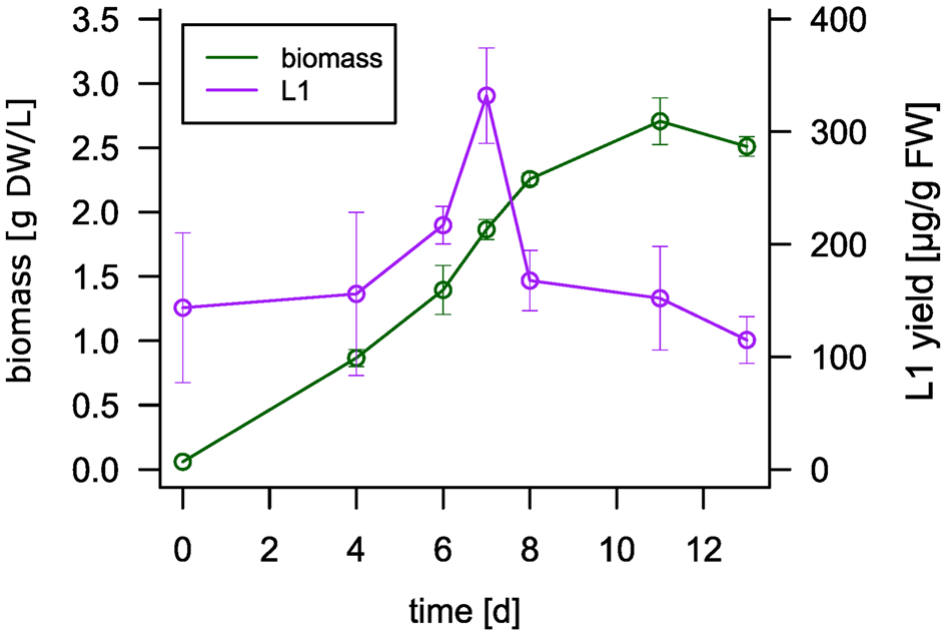
Analysis of biomass accumulation and L1 production of line CTP-pL1 30 in a 5 L-photobioreactor. The bioreactor was inoculated with 60 mg dry weight (DW)/L and monitored over a course of 13 days. On day 4, 5 µM auxin (NAA) was added. L1 production peaked at day 7 at 332 µg/g fresh weight (FW). Biomass and L1 measurements are based on n ≥ 3 technical replicates. Production levels of L1 were quantified via ELISA.

Biomass increased from initially 60 mg DW/L to around 870 mg DW/L at day 4 (Fig. 3). L1 production remained constant during the first 4 days, in the range of the yield observed for flasks. Upon addition of the phytohormone auxin (NAA) on day 4, L1 levels increased on day 6 and peaked at day 7 at 332 µg/g FW, 130% higher as compared to day 0. Afterwards, L1 production dropped sharply to levels prior to auxin addition. Biomass continued to accumulate until day 11, after which it dropped slightly. These conditions, including day 7 as the ideal harvesting time point, were used for further L1 production.

### Two-step purification removes bulk of cellular proteins

Purification of L1 was performed using ammonium-sulphate (AMS) precipitation, followed by cation exchange (CaEx) chromatography. While AMS removes the bulk of cellular proteins, CaEx removes residual proteins and concentrates the sample.

We tested the efficacy of 40, 45 and 50% (w/v) AMS to precipitate L1 from an extract containing the soluble protein fraction from CTP-pL1 30 plant material produced in the bioreactor. We found 45% AMS to be sufficient to effectively remove L1 from the supernatant, whereas at 40% AMS small amounts of L1 remained in the supernatant and at 50% AMS higher amounts of cellular protein were precipitated together with L1 (Supplementary Figure S7). In preparation for chromatographic purification, the precipitated proteins were resuspended in CaEx binding buffer and dialyzed overnight in binding buffer to remove residual AMS. Cloudiness of samples indicated a reduced solubility of precipitated proteins, and undissolved matter was removed by filtration. The relative amount of L1 and host protein was evaluated via western blots and Coomassie-staining, respectively (Supplementary Figure S7). Samples after filtration showed a slight reduction in host protein content, but, except for the sample at 40% AMS, no reduction in L1. Overall, precipitation at 45% AMS was selected, which is in line with purifications of yeast-produced L1 (Park et al. 2008; Kim et al. 2010).

**Fig. 4:**
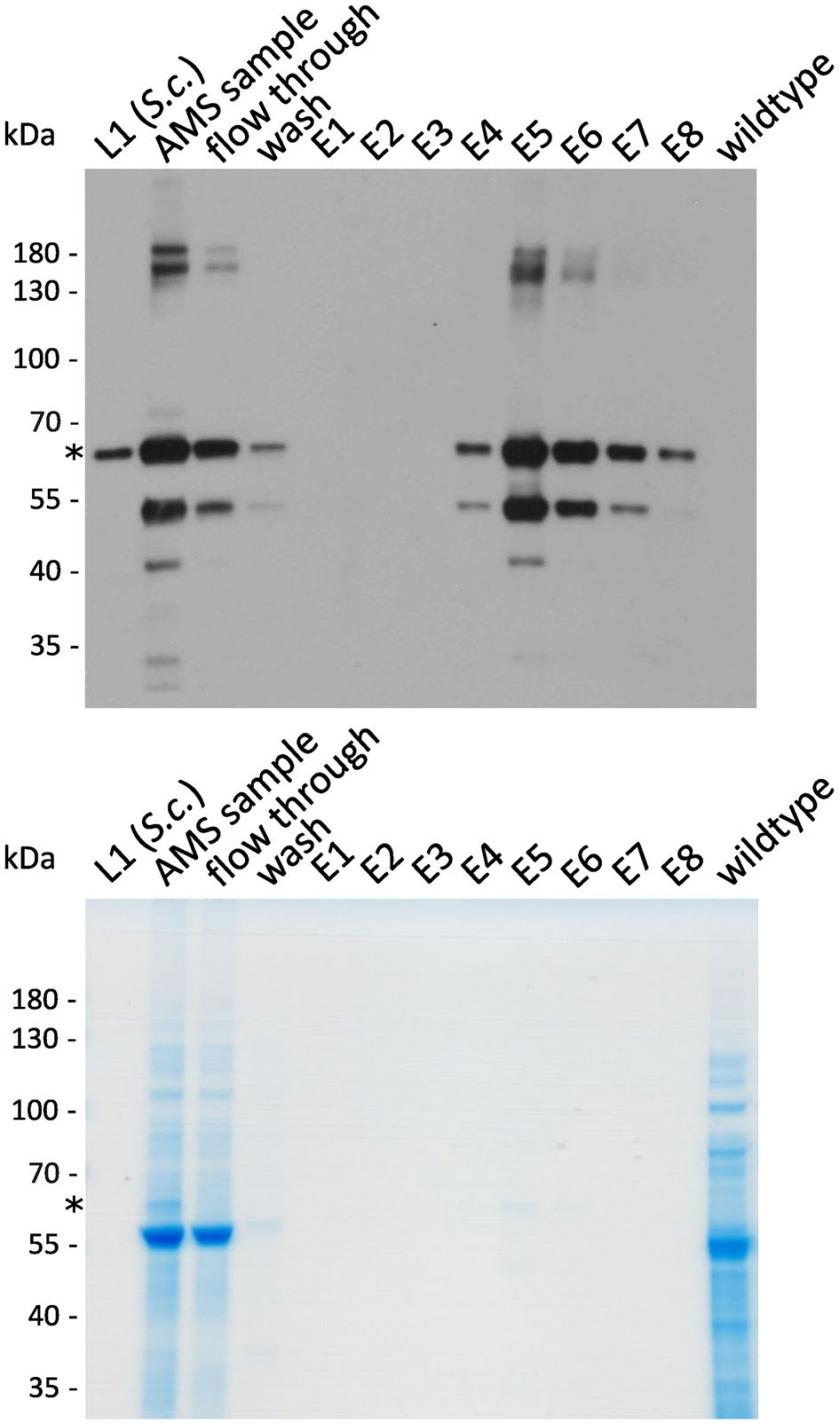
Two-step purification of L1 removes bulk of cellular protein. Analysis of L1 and residual soluble protein during purification under reducing conditions via western blot and Coomassie-stained SDS-PAGE (12% Bis–Tris gel). After precipitation with 45% (w/v) AMS, the filtered sample was subjected to cation exchange chromatography (CaEx) for final purification. Column-bound L1 eluted at 1 M NaCl in fractions E4–E8. The asterisk in the Western Blot and Coomassie-stained SDS-PAGE marks the band referring to HPV-16 L1. 50 ng *S. cerevisiae*-produced L1 served as a positive control and Physcomitrella wildtype as a negative control.

Next, CaEx chromatography was used to remove residual cellular proteins. The bulk of cellular proteins did not bind to the column and was detected in the flow through (Fig. 4, Supplementary Figure S8). Notably, a large amount of L1 did not bind to the column and was detected in the flow through. Different binding buffer compositions were tested to increase L1 binding to the column, e.g. lowering the pH from 7.2 to 6.8, lowering the concentration of NaCl from 0.137 M to 0.068 M, addition of 5% (v/v) glycerol, and re-loading of flow-through fraction. Nevertheless, a large part of L1 did not bind to the column and was found in the flow through. During column wash with 5 mL at 0.3 M NaCl, small amounts of contaminating proteins and L1 were washed off the column. For elution, NaCl was increased to 1 M NaCl and 0.5 mL fractions were collected, in total 4 mL. L1 eluted from the column in fractions E4–E8 (Fig. 4), which were pooled for further analysis.

### Capsomers and VLPs

Next, we submitted the moss-produced L1 to a dis- and reassembly protocol, as it yields more uniform and stable VLPs, which are less prone to aggregation (McCarthy et al. 1998; Mach et al. 2006). First, pooled elution fractions were dialyzed at low salt concentrations, high pH and DTT, that results in the disassembly of VLPs or other multi-pentameric L1-based structures into capsomers. Second, samples were dialyzed at high salt, low pH and in the absence of reducing agent, that leads to the reassembly of capsomers into VLPs.

To assess the conformation of L1 throughout the process, dot blots were performed using different monoclonal antibodies. CamVir1 detects a linear epitope in both denatured and native L1 and served as a control. In contrast, H16.V5 detects only conformational epitopes in both, L1 capsomers and VLPs, but not monomeric or denatured L1.

**Fig. 5:**
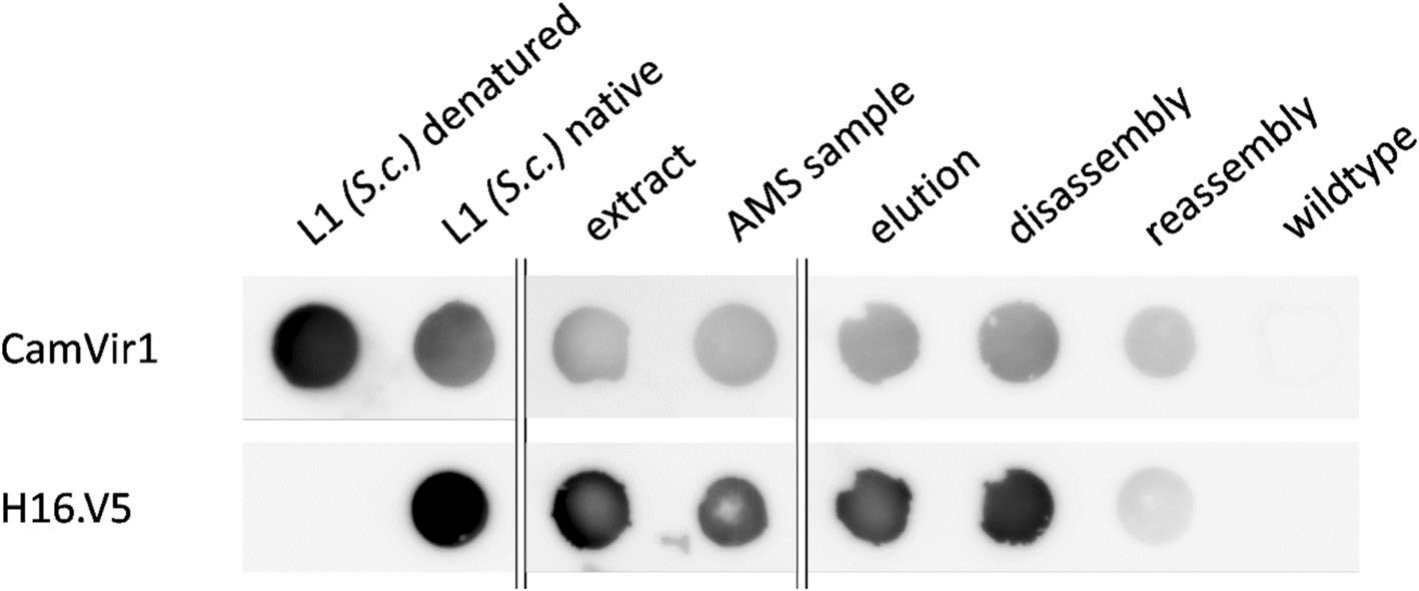
Moss-produced HPV-16 L1 assembles into capsomers, which are stable throughout purification. Dot blots were performed using two different antibodies to analyse the formation of capsomers in yeast-produced L1 and samples from CTP-pL1 30 purification (extract, after AMS precipitation, elution) and dis- and reassembly. The CamVir1 antibody detects a linear epitope in both denatured and native L1. The neutralizing antibody H16.V5 detects specifically conformational epitopes in L1 capsomers and VLPs. Positive H16.V5 signals indicate HPV-16 L1 monomers to assemble at least into capsomers, in vivo or after extraction, which are stable throughout the purification. The experiment only allows qualitative but not quantitative comparison since sample concentrations were adjusted individually. ‘L1 denatured’ was incubated at 95 °C for 5 min, all other samples were kept at 4 °C. A wildtype sample was used as a negative control.

The dot blot showed the presence of L1 in all CTP-pL1 30-derived samples throughout the purification process, as well as yeast-derived denatured and native L1 (Fig. 5). In comparison, H16.V5 detects L1 in the native positive control and in all CTP-pL1 30-derived samples, indicating the presence of capsomers, if not VLPs, already in the raw extract. Further, conformational epitopes seem to be stable throughout AMS precipitation, CaEx chromatography, disassembly, and reassembly. However, the reassembly sample exhibits a weaker signal for H16.V5 as compared to CamVir1.

**Fig. 6:**
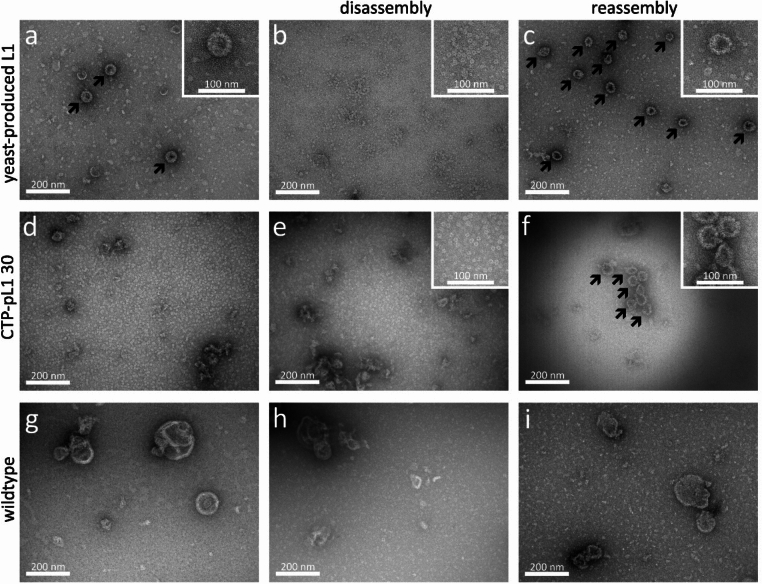
Analysis of dis- and reassembled VLPs from moss and yeast via transmission electron microscopy (TEM). Yeast-derived L1 was analysed untreated (**a**), after disassembly (**b**), and after reassembly (**c**) via TEM. CTP-pL1 30 (**d**–**f**) and wildtype (**g**–**i**) extracts were purified via AMS precipitation and CaEx chromatography (**d**,** g**), after disassembly (**e**,** h**), and after reassembly (**f**, **i**). Untreated yeast-derived VLPs (**a**) disintegrate into capsomers upon disassembly (**b**) and reshape into VLPs upon reassembly treatment (**c**). TEM shows the presence of moss-derived HPV-16 L1 capsomers in the elution (**d**) and disassembly sample (**e**) and the assembly into VLPs in the reassembly sample (**f**). Capsomer and VLP-like structures are absent from the wildtype control samples. VLPs are indicated by black arrows.

The assembly of moss-produced L1 into VLPs was verified with Transmission Electron Microscopy (TEM). As a control, yeast-produced L1 was analysed without prior treatment, after disassembly, and after reassembly (Fig. 6a–c). In the untreated sample, particles of approx. 60 nm in size were detected. After disassembly, structures of 5–10 nm in size were observed, corresponding in size and appearance to capsomers observed for yeast-derived L1 (McCarthy et al. 1998). VLPs reshape upon reassembly treatment, demonstrating the successful dis- and reassembly of yeast-derived L1 VLPs. Notably, reassembled VLPs are heterogenous in size and shape, despite the controlled dis- and reassembly (Fig. 6).

To analyse moss-produced L1 assembly into VLPs, samples were taken from the pooled CaEx chromatography elution fractions, after disassembly, and after reassembly (Fig. 6d–f).

In the elution sample, no VLPs but capsomeric structures were observed, comparable to those in the disassembled yeast sample, which are not present in the corresponding moss wildtype control (Fig. 6g–i). The abundance of capsomers in the elution fractions was surprising given the fact that the elution buffer has a high ionic strength, which favours reassembly. However, before column loading, the sample was dialyzed overnight in binding buffer, which favours disassembly. The exposure to high ionic strength during elution might have been too short to allow VLP assembly. In addition, both binding and elution buffer contained DTT, which induces disassembly (McCarthy et al. 1998; Mach et al. 2006). The disassembled CTP-pL1 30-derived sample depicted capsomers as seen in the elution fraction, and for disassembled yeast-derived L1 (Fig. 6). Figure 6f displays fully assembled, moss-derived HPV-16 L1 VLPs. Again, VLPs were heterogenous in size and shape, as seen for reassembled yeast-derived VLPs. Moss-produced VLPs tended to aggregate, which was not observed for reassembled yeast-derived VLPs, but is common for HPV-16 L1 VLPs (Shi et al. 2005). Aggregation might also explain the relatively low signal for the conformational antibody H16.V5 in the sample of reassembled moss-derived VLPs (Fig. 5). No structures resembling VLPs in size and shape were detected in wildtype samples (Fig. 6).

## Discussion

The goal of this study was to gain proof-of-concept for the stable production of virus-like particles (VLPs) in the moss plant Physcomitrella by producing HPV-16 L1 VLPs. For this purpose, we generated a coding sequence (CDS) optimized for expression of HPV-16 L1 in Physcomitrella. We further characterized five chloroplast transit peptides (CTPs) and confirmed the localization of L1 in chloroplasts. In addition, we demonstrated chloroplast localization to yield higher L1 levels than targeting the protein to nuclei or cytoplasm. Production in photobioreactors yielded more than 0.3 mg L1/g FW, which is in the range of other plant-based expression platforms. We established a purification protocol and demonstrated the successful assembly of moss-produced HPV-16 L1 VLPs.

In tobacco plants (*Nicotiana benthamiana*), various strategies were explored for increasing production yields of HPV-16 L1 VLPs, one of which is optimization of codon usage (Maclean et al. 2007; Hitzeroth et al. 2018). Hitzeroth et al. (2018) found that codon optimization of L1 improved transcription, rather than translation. This is due to RNA motifs in the L1 CDS, which post-transcriptionally regulate the virus life cycle and lead to pre-mRNA degradation (Mori et al. 2006; Zhao and Schwartz 2008). It is argued that codon optimization removes those motifs, thereby increasing mRNA and protein levels, irrespective of whether the CDS was optimized for expression in humans, tobacco (Hitzeroth et al. 2018) or Physcomitrella (this study). Our Physcomitrella-optimized CDS has a GC content of 62.85% as compared to hL1 with 62.25% (Maclean et al. 2007) and the wildtype sequence with 38.27% (Varsani et al. 2003). Combined with the removal of microRNA binding sites and heterosplice sites, according to established protocols (Top et al. 2021), we obtained the Physcomitrella-optimized pL1 CDS. We argue that pL1 addresses the issues of L1 intrinsic degradation motifs as well as Physcomitrella-specific posttranscriptional degradation.

Besides codon optimization, research focused on localization signals and transit peptides. The most common strategy is sequestration to chloroplasts, either via nuclear transformation and posttranslational import into chloroplasts (Maclean et al. 2007; Matic et al. 2011; Pineo et al. 2013; Zahin et al. 2016; Hitzeroth et al. 2018; Naupu et al. 2020; Muthamilselvan et al. 2023) or via transplastomic lines (Fernández-San Millán et al. 2008; Lenzi et al. 2008). Here, we experimentally characterized five Physcomitrella CTPs, of which we used one (CTPc5) for sequestration of L1 to chloroplasts. Employing the HPV-16 native NLS, L1 was targeted to nuclei, whereas the removal of the NLS resulted in cytoplasmic L1. Earlier studies confirmed functionality of the NLS in mammalian cells (Leder et al. 2001; Bissa et al. 2015). However, the NLS failed to localize L1 to nuclei in tobacco (Šmídková et al. 2010). Our observation of the HPV-16 native NLS targeting L1 to nuclei in Physcomitrella is another example for a conserved cross-kingdom functionality of localization signals between moss and mammals (Gitzinger et al. 2009).

Western blots of L1-producing Physcomitrella lines showed a variety of bands, both smaller and larger than the expected 56 kDa or 54 kDa. A comparable banding pattern was observed when expressing L1 in tobacco chloroplasts (Fernández-San Millán et al. 2008). Likewise, yeast-produced L1 exhibited bands with the size of L1 dimers and trimers (Kim et al. 2007, 2010). L1 dimers and trimers are based on inter-capsomeric disulfide bridges which stabilize virions and VLPs (Sapp et al. 1998; Buck et al. 2005, 2013). McCarthy et al. (1998) resolved L1 multimers with DTT. Likewise, Buck et al. (2005) demonstrated absence and presence of L1 dimers and trimers in a comparison of reducing and non-reducing SDS-PAGE and western blots, respectively. An investigation of cysteine 175 and cysteine 428 L1 mutants resulted in the absence of bands at 130 kDa and 180 kDa, thereby linking disulfide bridge-based dimers and trimers to the observed bands (Buck et al. 2005). Surprisingly, reducing agents such as DTT, ascorbic acid or β-mercaptoethanol did not result in the disappearance of these bands in our and other studies (Kim et al. 2007, 2010; Fernández-San Millán et al. 2008). This indicates a tolerance of L1 dimers and trimers to reducing agents or a different origin of multimerization observed at 130 kDa and 180 kDa. Castells-Graells and Lomonossoff (2021) observed multimerization of *Nudaurelia capensis* omega virus α-peptide produced in tobacco, irrespective of DTT, but not when produced in insect cells. These authors hypothesize that cross-linkage of α-peptide occurs during extraction and purification and occurs in plant-based expression systems. Regarding the observed bands below 56 kDa, proteolytic degradation was proposed in other studies observing the same phenomenon (Buck et al. 2005; Fernández-San Millán et al. 2008). In tobacco, a shift from bands above 100 kDa in young leaves to more prominent bands at 45 kDa and 20 kDa in older leaves indicated proteolytic degradation of L1 over time (Fernández-San Millán et al. 2008). In our study, bands at 45 kDa are most prominent in pL1Δ22 lines with cytoplasmic L1 and less prominent for plastidic L1. This might result from organelle-specific proteases (Coates et al. 2022).

In Physcomitrella, combining the full-length L1 with a CTP significantly increases L1 yield as compared to full length L1 or L1 without NLS, which is in line with findings in tobacco (Maclean et al. 2007; Zahin et al. 2016). In our study, L1 yields above 0.3 mg/g FW were achieved in the photobioreactor. In tobacco, yields of 0.1–1.2 mg/g FW (Maclean et al. 2007; Pineo et al. 2013; Zahin et al. 2016; Hitzeroth et al. 2018) and up to 3 mg/g FW in transplastomic lines have been reported (Fernández-San Millán et al. 2008). The success of targeting L1 to chloroplasts over localization to other organelles may have several reasons. One is that plastid L1 may be protected from proteases absent in chloroplasts (van Wijk 2024). Besides, CTPs also act on the transcriptional level. Upon expression in tobacco, chimeric L1/L2 transcripts containing a CTP were more abundant than transcripts lacking a CTP, and consequently yielded more protein (Hitzeroth et al. 2018). So far, it is unclear whether this difference is due to enhanced transcription, increased mRNA half-life or changes in mRNA secondary structure.

For purification of L1 from Physcomitrella extracts, we employed a combination of AMS precipitation and CaEx chromatography. We found 45% AMS to be sufficient to efficiently precipitate L1. Studies from yeast (Park et al. 2008; Kim et al. 2010) and tobacco (Zahin et al. 2016) also used 45% AMS, whereas sometimes 50% AMS was used (Kim et al. 2018). CaEx chromatography showed that a large part of L1 does not bind to the column. Likewise, Zahin et al. (2016) reported tobacco-produced L1 to bind poorly to CaEx columns*.* Kim et al. (2010) purified yeast-produced L1 by either CaEx or heparin chromatography. CaEx chromatography showed unbound L1 in the flow through, as observed in our study, whereas no L1 was found in the flow through employing heparin chromatography. Pineo et al. (2013) produced L1/L2 chimaeras in tobacco and experienced loss of L1 and insufficient removal of contaminating proteins using heparin chromatography. Heparin is a highly sulphated form of heparan sulfate, a proteoglycan found on host cell surfaces and a requirement for HPV virion attachment and infection (Knappe et al. 2007). However, a prerequisite for heparin binding is the assembly of L1 to capsomers or VLPs (Rommel et al. 2005), meaning monomers and not properly folded L1 are lost. Overall, chromatography yields for VLPs differ and are influenced by different factors, including the production host, the used materials and buffers, and purification prior to chromatography, making it difficult to compare purification strategies across publications. Here, we achieved to remove the bulk of cellular proteins from moss extracts, but experienced a substantial loss of L1 in the flow through. To address this, further optimization of purification might be achieved by combinations with other methods such as heparin chromatography. Our dot blot analysis confirmed the presence of intact capsomers or VLPs in extracts and after AMS precipitation, suggesting heparin chromatography as an option for the second step in purification.

TEM analysis of purified and reassembled CTP-pL1 samples shows VLPs of approx. 60 nm in size. These nanoparticles assembled from capsomers as a result of the reassembly treatment. The absence of VLPs in the elution fractions does not necessarily indicate that dis- and reassembly treatment is a prerequisite for VLP formation from moss-produced L1, but rather that the buffer conditions did not favor assembly after elution. To the best of our knowledge, this is the first report of VLPs produced in a non-vascular plant and of in vitro reassembly of HPV-16 L1 VLPs from capsomers produced in any plant system. The VLPs are heterogenous in shape and size. The same is true for yeast-derived VLPs after reassembly, whereas untreated VLPs were not heterogenous. This is surprising, since controlled dis- and reassembly of VLPs can enhance stability and uniformity (McCarthy et al. 1998; Mach et al. 2006; Shi et al. 2007). Besides high ionic strength and low pH buffer, inter-pentameric disulfide bridges positively influence VLP stability and uniformity (Buck et al. 2005). This could be achieved through prolonged incubation of cleared cell lysates before purification or addition of oxidizing agents, which accelerate disulfide bridge formation. However, prolonged incubation causes aggregation of HPV-16 L1 VLPs, as observed here, but could be addressed by improved formulation (Shi et al. 2005).

## Conclusion

While the moss plant Physcomitrella is an established production host for biopharmaceuticals, we aim to expand its application to other markets such as the cosmetics industry (Munoz et al. 2024) and material sciences at the interface with biomedicine (Milferstaedt et al. 2025; Ramezaniaghdam et al. 2025). In this context, the present study serves as a proof-of-concept for the production of a relatively simple but medicinally relevant VLP in Physcomitrella. Future efforts may focus on more complex eVLPs that require proper N-glycosylation or complex folding and assembly; a specialty niche well suited for the moss-based expression platform.

## Materials and methods

### Plant material

Physcomitrella (new species name: *Physcomitrium patens* (Hedw.) Mitt.; IMSC accession number 41269), was cultivated according to Reski and Abel (1985) and Decker et al. (2015).

### Optimization of HPV-16 L1 coding sequence

The coding sequence (CDS) of h(human codon-optimized)L1 (GenBank acc. no. DQ067889, Maclean et al. 2007) was used for optimization using physCO (https://www.plant-biotech.uni-freiburg.de/tools/physco/; Top et al. 2021). Putative alternative splicing motifs AGGT were identified via search function. MicroRNA-binding sites were predicted via psRNATarget with the library of 280 published Physcomitrella microRNAs and the default settings *Schema V2* (2017 release) (http://plantgrn.noble.org/psRNATarget/; Dai and Zhao 2011; Dai et al. 2018). Alternative splice sites and microRNA-binding sites were removed and substituted by synonymous codons based on http://www.kazusa.or.jp/codon/. Sequence editing was done in Benchling (https://www.benchling.com/).

### Expression vectors

Chloroplast transit peptides (CTPs) were selected from a list of proteins with experimentally confirmed cleavage of a predicted CTP (Hoernstein et al. 2024). CTP-eGFP vectors were constructed based on a vector containing an eGFP CDS with an N-terminal linker (sequence: GGGGGA) under control of the PpActin5 promoter (Weise et al. 2006) and nos terminator (Hoernstein et al. 2023). CTP candidates were amplified from Physcomitrella cDNA and inserted at the N-terminus of the linker using Gibson Assembly (Gibson et al. 2009). The Citrine-L1 fusion constructs are based on a vector containing the PpActin5 promoter, nos terminator and citrine CDS (Wiedemann et al. 2018). The CDS of pL1, pL1Δ22 and CTP-pL1 were amplified using Phusion™ polymerase (Thermo Fisher Scientific, Waltham, USA), cloned into the pJet1.2 backbone (Thermo Fisher Scientific) and ligated into the expression vector using T4 Ligase (Thermo Fisher Scientific), via* Xho*I and *BamH*I sites. The pL1 expression vectors were constructed based on a vector containing the PpActin5 promoter, nos terminator and hpt cassette (Ruiz-Molina et al. 2022a). The pL1 sequence was synthesized by Genewiz (Leipzig, Germany). For constructs pL1 and pL1Δ22, the pL1 CDS was amplified using Phusion™ polymerase (Thermo Fisher Scientific) and cloned as described above. For construct CTP-pL1, the pL1 and CTPc5 CDS were amplified and inserted into the backbone using Gibson Assembly. Vectors were sequenced by Eurofins Genomics (Ebersberg, Germany). Vector maps are compiled in Supplementary Figure S1. Primers were synthesized by Eurofins Genomics and are compiled in Supplementary Table S1.

### Protoplast preparation and transformation

Physcomitrella protoplasts were prepared and transformed based on existing protocols (Hohe and Reski 2002; Schween et al. 2003; Hohe et al. 2004; Decker et al. 2015). Transformed protoplasts were incubated at 22 °C for 24 h in the dark and subsequently for 72 h at a 16/8-h light/dark photoperiod and light intensity of 50–70 μmol m^−2^ s^−1^ before plating on KnopME + 25 mg/L hygromycin agar plates covered with cellophane for two weeks. Surviving colonies were transferred to non-selective conditions for two weeks before a second selection on KnopME + 50 mg/L hygromycin.

### Fluorescence microscopy

Protoplasts were analysed 2–3 days after transfection with an AxioCam MRc5 camera on an Axioplan2 stereo microscope (Zeiss, Oberkochen, Germany). Pictures were taken with a 40× objective or a 63× objective with immersion oil. A FITC filter was used for eGFP, a rhodamine filter for chlorophyll autofluorescence, and a FITC_LP filter for the combination of the two. Images were edited with GIMP 2.10.22 and ImageJ. For confocal microscopy, an AXIO Observer.Z1 Inverted Fluorescence Motorized Phase Contrast Microscope Pred 7 with the objective Plan-Apochromat 63x/1.4 Oil DIC M27 (Zeiss) was used. EGFP signals were detected at 488/509 nm, and chlorophyll autofluorescence at 587/610 nm. Images were edited in ZEN 3.5 (Blue edition, ZEN Lite), ImageJ, and GIMP 2.10.22.

For analysis of subcellular targeting, an inverted confocal laser scanning microscope LSM 880 with the objective LD LCI Plan-Apochromat 40x/1.2 H_2_O autocorr and FastAiryScan mode (Zeiss) was used. Citrine signals were detected at 488/516 nm, and chlorophyll autofluorescence at 561/595 nm. Images were edited in ZEN 3.9 (Blue edition) and processed via deconvolution using default setting and orthogonal projection using maximum intensity projection.

### Biomass accumulation

Flasks containing 30 mL KnopME medium were inoculated at a density of 60 mg dry weight (DW)/L and cultured for 4 weeks at the conditions described above. Biomass DW was measured by filtering 3 × 10 mL of culture through miracloth and subsequent drying at 105 °C for 2 h. Biomass accumulation was analysed by linear regression analysis. Slopes were compared using ANCOVA and post hoc multiple comparisons test (TukeyHSD). The analysis was conducted in RStudio (version 4.2.2) and GraphPad Prism (version 8.1).

### Bioreactor operation

Stirred tank bioreactors (Getinge, Sweden) were inoculated at 60 mg DW/L moss density in 5 L KnopME. The light intensity was set to 160 µmol m^−2^ s^−1^ and increased to 350 μmol m^−2^ s^−1^ after 4 days. At day 4, auxin 1-Naphtalene acetic acid (NAA, Sigma, St. Louis, USA) dissolved in 0.5 M KOH was added to a final concentration of 5 µM (Ruiz-Molina et al. 2022b). Throughout the bioreactor run, the pH was kept at pH 5.8, temperature at 22 °C, and aeration at 0.3 vvm (volume air per volume medium per minute) with 2% CO_2_. The culture was continuously agitated with a pitched 3 blade impeller at 500 rpm.

### Protein extraction, purification and VLP assembly

For small scale extraction, 30–60 mg fresh weight (FW) of vacuum-filtered moss material was combined with a glass and a steel bead (each 3 mm in diameter) in a 2 mL reaction tube, frozen in liquid nitrogen and the tissue lysed using a tissue lyser for 2 min at 28 Hz. Homogenized material was resuspended in PBS (2.7 mM KCl, 10 mM Na_2_HPO_4_, 1.8 mM KH_2_PO_4_, 0.137 M NaCl, pH 7.2), 0.01% (v/v) Tween20, and protease inhibitor (P9599, Sigma) in a ratio 3 mL/g. Samples were placed in an ice-cold sonication bath for 15 min, centrifuged for 30 min at 14,000×*g* and 4 °C, and the supernatant taken off for further analysis.

For large scale protein extraction, 5 g FW of vacuum-filtered moss material were frozen in liquid nitrogen and ground to a fine powder. The material was combined with 22 mL PBS and 0.01% (v/v) Tween20, 50 mM DTT and 100 µL protease inhibitor. The mixture was homogenized using an ULTRA-TURRAX (IKA, Staufen, Germany) at 10,000 rpm for 10 min on ice. Next, the extract was treated with a Q500 sonicator using a CL-334 converter (QSonica, Newtown, USA) at amplitude 55%, 10 s on & 40 s off for 20 min at 8 °C. Debris was removed by two centrifugations at 4500×*g* for 10 min and 20,000×*g* for 20 min, both at 4 °C. The sample was combined with solid (NH_4_)_2_SO_4_ to a final concentration of 45% (w/v) and proteins precipitated under stirring for 1 h at 8 °C. Amounts of (NH_4_)_2_SO_4_ were calculated using an online tool (https://files.encorbio.com/protocols/AM-SO4.htm) (EnCor Biotechnology Inc., Gainesville, USA). Precipitated proteins were pelleted by centrifugation at 12,000×*g* for 10 min at 8 °C. Pellets were dissolved in 4 mL cation exchange (CaEx) chromatography binding buffer (2.7 mM KCl, 10 mM Na_2_HPO_4_, 1.8 mM KH_2_PO_4_, 0.068 M NaCl, 0.01% (v/v) Tween 20, 5% (v/v) Glycerol, 5 mM DTT, pH 6.8) and dialyzed (20k MW cut off, 88405, Thermo Fisher Scientific) overnight against the same buffer at 8 °C. The sample was diluted to a final volume of 10 mL using binding buffer, and passed through a 0.22 µm filter (Roth, Karlsruhe, Germany) before loading onto a 1 mL HiTrap SP HP cation exchange column (Cytiva, Marlborough, USA), connected to an ÄKTA™ start (Cytiva). The column was washed with 5 column volumes (CV) of 0.3 M NaCl (based on binding and elution buffer) and L1 eluted via 4 CV of elution buffer (2.7 mM KCl, 10 mM Na_2_HPO_4_, 1.8 mM KH_2_PO_4_, 1 M NaCl, 0.01% (v/v) Tween 20, 5% (v/v) Glycerol, 5 mM DTT, pH 6.8).

Eluted fractions containing L1 were pooled and dialyzed against disassembly buffer (22.7 mM KCl, 10 mM Na_2_HPO_4_, 1.8 mM KH_2_PO_4_, 0.068 M NaCl, 0.05% (v/v) Tween20, 5 mM DTT, pH 8.5) overnight at 8 °C with 3× buffer exchange. Samples were concentrated 5× using a vacuum concentrator (Concentrator plus, Eppendorf, Hamburg, Germany). For reassembly, samples were dialyzed against reassembly buffer (22.7 mM KCl, 10 mM Na_2_HPO_4_, 1.8 mM KH_2_PO_4_, 1 M NaCl, 0.05% (v/v) Tween20, pH 6) overnight at 8 °C with 3× buffer exchange.

### SDS-PAGE and western blot

SDS-PAGE and western blots were performed based on Bohlender et al. (2022). Two systems were used for protein separation, either 7.5% polyacrylamide gels (Mini-PROTEAN^®^ TGX™ Precast Gels, Rio-Rad, Munich, Germany) in TGS buffer at 120 V, or 4–12% Bis–Tris gels (mPAGE™ Precast Gels, Merck, Darmstadt, Germany) in MOPS buffer at 150 V. As first antibody served 1:5000 diluted CamVir1 (Thermo Fisher Scientific), as second antibody peroxidase-linked rabbit anti-mouse secondary antibody (Cytiva) diluted 1:12,500. For dot blots, protein samples were blotted on a PVDF membrane (Cytiva) using a filtration manifold kit (SRC-96/1, Schleicher&Schuell, Dassel, Germany). Antibody H16.V5 (AntibodySystems, Schiltigheim, France) was diluted 1:5000. Dot blots were developed as described for western blots. Yeast-produced HPV-16 L1 (Abcam, Cambridge, UK) was used as a positive control.

### L1 quantification

The ELISA protocol was based on Studentsov et al. (2002). In short, 96-well microtiter plates (Greiner, Kremsmünster, Austria) were coated with 1:500 CamVir1 antibody (Thermo Fisher Scientific) in PBS overnight at 8 °C. Wells were washed twice with washing buffer (1× PBS, 0.05% (v/v) Tween20) and blocked with blocking buffer (1× PBS, 1% (w/v) polyvinyl alcohol MW 47,000, 0.05% (v/v) Tween20) for 3 h at 37 °C. After 3× washing, samples were diluted in blocking buffer and incubated in the wells for 1.5 h at 37 °C. Wells were washed 3× before adding 1:1000 diluted polyclonal anti-HPV-16 L1 antibody from rabbit (Cusabio, Houston, USA) for 1 h at 37 °C. After 5× washing, wells were incubated with 1:1000 diluted anti-rabbit HRP-conjugated antibody NA934 (Cytiva) for 1 h at 37°C. Wells were washed 3× before adding 100 µL TMB substrate (Thermo Fisher Scientific). The reaction was stopped with 50 µL of 2 M H_2_SO_4_. Absorbance was measured at 450/595 nm for reference. The signal of a wildtype sample was subtracted to account for background signal.

### Transmission electron microscopy

The TEM protocol was based on Abel et al. (1989). Negative staining of purified VLPs and moss extracts was performed using 300 Mesh glow discharged formvar/carbon-coated copper grids (Electron Microscopy Sciences, Hatfield, USA). Five µL of sample were applied on a grid and incubated for 5 min at RT. Grids were washed 3× with water and stained with 2% (w/v) uranyl acetate. After 30 s, uranyl acetate was removed with a filter paper, grids were air dried, and inspected with a Hitachi HT7800 TEM (Tokyo, Japan) coupled to a Xarosa CMOS camera (Emsis, Münster, Germany).

## Supplementary Information

Below is the link to the electronic supplementary material.

Supplementary file1 (DOCX 4132 KB)

## Data Availability

Nucleotide sequence data was submitted to GenBank under accession numbers PV029542 (pL1) and PV068547(CTPc5). Moss lines and vectors were submitted to the International Moss Stock Centre (IMSC) under accession numbers 40963 (pL1 5A), 40964 (pL1 23A), 40965 (pL1 47B), 40966 (pL1Δ22 8A), 40967 (pL1Δ22 11B), 40968 (pL1Δ22 102B), 40969 (CTP-pL1 30), 40970 (CTP-pL1 103), 40971 (CTP-pL1 143), 1840 (pAct5_linker_eGFP_35ST_hpt), 2038 (pAct5_CTP_linker_eGFP_35ST_hpt), 1193 (pAct5_Citrine_nosT_hpt), 2249 (pAct5_pL1-Citrine_nosT_hpt), 2250 (pAct5_pL1Δ22-Citrine_nosT_hpt), 2251 (pAct5_CTP-pL1-Citrine_nosT_hpt), 1997 (pAct5_pL1_35ST_hpt), 1998 (pAct5_pL1Δ22_35ST_hpt) and 2048 (pAct5_CTP-pL1_35ST_hpt).
