# Supplementary material for: Production of human papillomavirus type 16 virus-like particles in Physcomitrella photobioreactors
Source: Plant Cell Rep. 2025 Sep 17;44(10):216. doi: 10.1007/s00299-025-03602-x (PMC12443937; doi:10.1007/s00299-025-03602-x)
Supplement: Supplementary file 1 — Supplementary file1 (DOCX 4132 KB) [file 299_2025_3602_MOESM1_ESM.docx]

**Supplementary Information**

**Production of human papillomavirus type 16 virus-like particles in Physcomitrella photobioreactors**

Paul Alexander Niederau^1^, Maria Caroline Weilguny^1,2^, Sarah Chamas^1^, Caitlin Elizabeth Turney^1^, Juliana Parsons^1^, Marta Rodríguez-Franco^3^, Sebastian N.W. Hoernstein^1^, Eva L. Decker^1^, Henrik Toft Simonsen^2,4^, Ralf Reski^1,5,6 *^

^1^ Plant Biotechnology, Faculty of Biology, University of Freiburg, Schänzlestr. 1, 79104 Freiburg, Germany

^2^ Department of Biotechnology and Biomedicine, Technical University of Denmark, 2800 Kongens Lyngby, Denmark

^3^ Cell Biology, Faculty of Biology, University of Freiburg, Schänzlestr. 1, 79104 Freiburg, Germany

^4^ Laboratoire de Biotechnologies Végétales appliquées aux Plantes Aromatiques et Médicinales, CNRS, UMR 5079, Université Jean Monnet, 42023 Saint-Etienne Cedex 2, France

^5^ Signalling Research Centre BIOSS and CIBSS, University of Freiburg, Schänzlestr. 18, 79104 Freiburg, Germany

^6^ Cluster of Excellence *liv*MatS @ FIT – Freiburg Centre for Interactive Materials and Bioinspired Technologies, University of Freiburg, Georges-Köhler-Allee 105, 79110 Freiburg, Germany

***Corresponding author:** ralf.reski@biologie.uni-freiburg.de

**Supplementary Figure S1 – Vector maps**


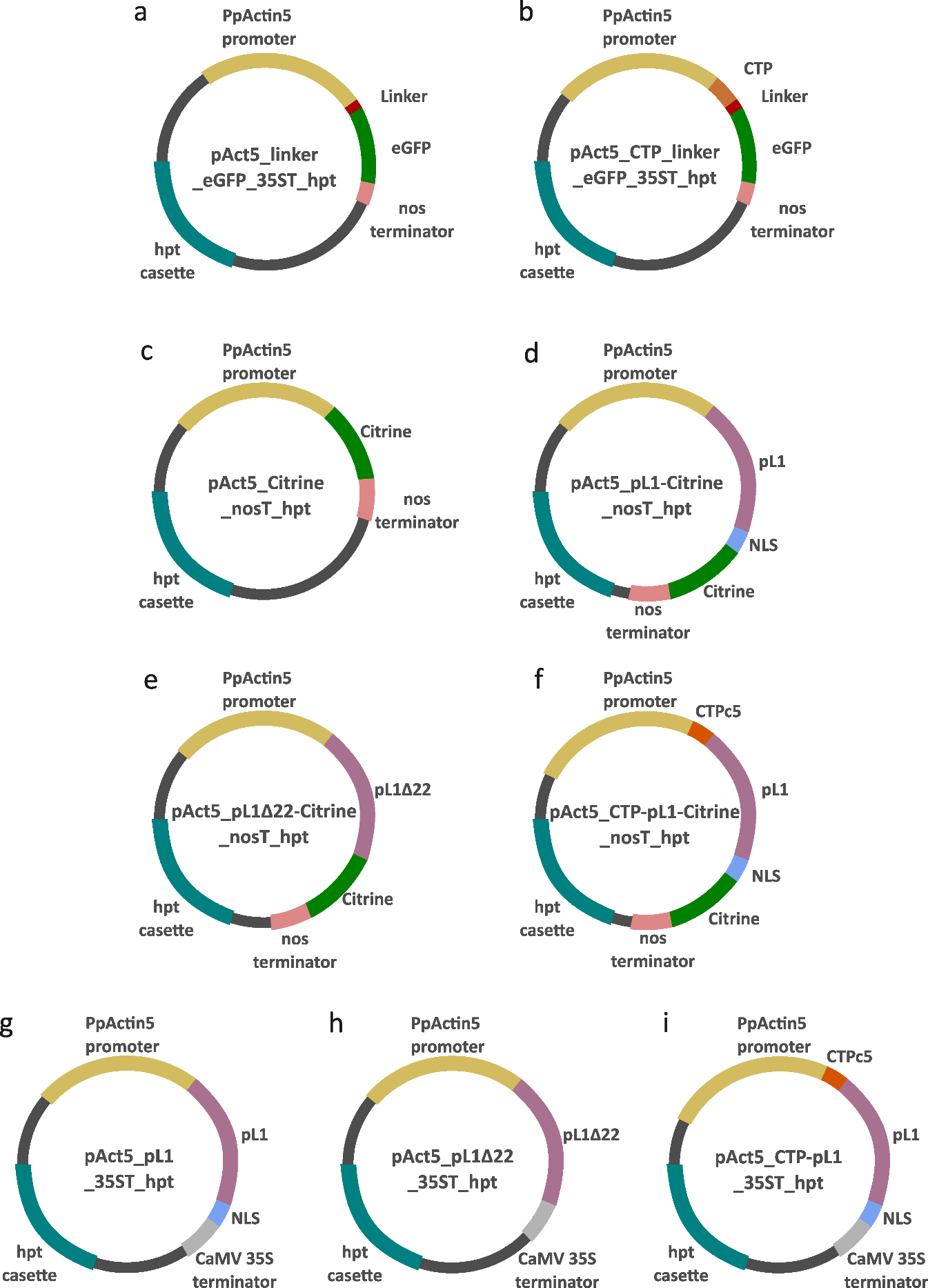


**List of all vectors used in this study.** Vectors **a** & **b** were used for the characterization of chloroplast transit peptides (CTPs). Vector **a** served as a control and was used as a cloning template for inserting different CTP candidates. Vectors **c**, **d**, **e** & **f** were used for the analysis of L1 subcellular targeting. Vector **c** served as a control and was used as a cloning template for inserting either pL1, pL1Δ22 or CTP-pL1 CDS (**d**, **e** & **f**, respectively). Vectors **g**, **h** & **i** were used for generation of stable transgenic Physcomitrella lines. The vectors contain either the full length pL1 including the native nuclear localization signal (NLS) (**g**), a truncated version lacking the NLS (**h**), or the full-length version including the native NLS and an N-terminal chloroplast transit peptide (**i**). All vectors contain the PpActin5 promoter (yellow), the CaMV 35S terminator (grey), and a hygromycin (hpt)(turquoise) resistance cassette for selection. Other elements are the GGGGGA linker (red), CTP (orange), NLS (blue) and eGFP or Citrine (green). Genetic elements are not depicted true to scale. Vector sizes: 7260 bp (**a**), 7461 bp (**b**), 7299 bp (**c**), 8802 bp (**d**), 8736 bp (**e**), 9021 bp (**f**), 7998 bp (**g**), 7932 bp (**h**), 8217 bp (**i**).

## **Supplementary Figure S2 - Generation of Physcomitrella-optimized (p)L1**

**HPV-16 hL1**

ATGTCCCTGTGGCTGCCCAGCGAGGCCACCGTGTACCTGCCCCCCGTGCCCGTGAGCAAGGTGGTGAGCACCGATGAGTACGTGGCCCGGACCAACATCTACTACCACGCCGGCACCTCCAGACTGCTGGCCGTGGGCCACCCCTACTTCCCCATCAAGAAGCCCAACAACAACAAGATCCTGGTGCCCAAGGTGAGCGGCCTGCAATACCGGGTGTTCAGAATCCACCTGCCCGACCCCAATAAGTTCGGCTTCCCCGACACCAGCTTCTACAACCCCGACACCCAGAGACTGGTGTGGGCCTGCGTGGGCGTGGAGGTGGGCAGAGGCCAGCCTCTGGGCGTGGGCATCAGCGGCCACCCTCTGCTGAACAAGCTGGACGACACCGAGAACGCCAGCGCCTACGCCGCCAACGCCGGCGTGGATAACAGAGAATGCATCAGCATGGACTACAAGCAGACCCAGCTGTGCCTCATCGGCTGCAAGCCCCCCATCGGCGAGCACTGGGGCAAGGGCAGCCCCTGCACCAACGTGGCCGTGAATCCTGGCGACTGTCCTCCCCTGGAACTCATCAACACCGTGATCCAGGACGGCGACATGGTGGACACCGGCTTCGGCGCCATGGACTTCACCACCCTCCAGGCCAATAAGAGCGAGGTGCCCCTGGACATCTGCACCAGCATCTGCAAGTACCCCGACTACATCAAGATGGTGAGCGAGCCCTACGGCGATAGCCTGTTCTTCTACCTGCGGCGGGAGCAGATGTTCGTGCGGCACCTGTTCAACAGAGCCGGCGCCGTGGGCGAGAACGTGCCCGACGACCTGTACATCAAGGGCAGCGGCAGCACCGCCAACCTGGCCAGCAGCAACTACTTCCCTACCCCCAGCGGCTCCATGGTGACCAGCGACGCCCAGATCTTCAACAAGCCCTACTGGCTCCAGAGAGCCCAGGGCCACAACAATGGCATCTGCTGGGGCAACCAGCTGTTCGTGACCGTGGTGGATACCACCCGGAGCACCAACATGTCCCTGTGCGCCGCCATCAGCACCAGCGAGACCACCTACAAGAACACCAACTTCAAGGAGTACCTGAGGCACGGCGAGGAGTACGACCTCCAGTTCATCTTCCAGCTGTGCAAGATCACCCTCACCGCCGACGTGATGACCTACATCCACAGCATGAACAGCACCATCCTGGAGGACTGGAACTTCGGCCTGCAGCCCCCTCCTGGCGGCACCCTGGAGGACACCTACAGATTCGTGACCAGCCAGGCCATCGCATGCCAGAAGCACACCCCTCCCGCCCCTAAGGAGGACCCCCTGAAGAAGTACACCTTCTGGGAGGTGAACCTGAAGGAGAAGTTCAGCGCCGACCTGGACCAGTTCCCTCTGGGCAGAAAGTTCCTGCTGCAAGCCGGCCTGAAGGCCAAGCCTAAGTTCACCCTGGGCAAGAGAAAGGCCACCCCCACCACAAGCAGCACCAGCACCACCGCCAAGCGGAAGAAGCGCAAGCTGTGA

**HPV-16 pL1**

ATGTCCCTGTGGCTGCCCAGCGAGGCCACCGTGTACCTGCCCCCCGTGCCCGTGAGCAAAGTGGTGAGCACCGATGAGTACGTGGCCCGGACCAACATCTACTACCACGCCGGCACCTCCAGACTGCTGGCCGTGGGCCACCCCTACTTCCCCATCAAGAAGCCCAACAACAACAAGATCCTGGTGCCCAAAGTGAGCGGCCTGCAGTACCGGGTGTTCAGAATCCACCTGCCCGACCCCAACAAGTTCGGCTTCCCCGACACCAGCTTCTACAACCCCGACACCCAGAGACTGGTGTGGGCCTGCGTGGGCGTGGAAGTGGGCAGAGGCCAGCCTCTGGGCGTGGGCATCAGCGGCCACCCTCTGCTGAACAAGCTGGACGACACCGAGAACGCCAGCGCCTACGCCGCCAACGCCGGCGTGGATAACAGAGAGTGCATCAGCATGGACTACAAGCAGACCCAGCTGTGCCTCATCGGCTGCAAGCCCCCCATCGGCGAGCACTGGGGCAAGGGCAGCCCCTGCACCAACGTGGCCGTGAACCCTGGCGACTGCCCTCCCCTGGAGCTCATCAACACCGTGATCCAGGACGGCGACATGGTGGACACCGGCTTCGGCGCCATGGACTTCACCACCCTCCAGGCCAACAAGAGCGAAGTGCCCCTGGACATCTGCACCAGCATCTGCAAGTACCCCGACTACATCAAGATGGTGAGCGAGCCCTACGGCGATAGCCTGTTCTTCTACCTGCGGCGCGAGCAAATGTTTGTGCGGCACCTGTTCAACAGAGCCGGCGCCGTGGGCGAGAACGTGCCCGACGACCTGTACATCAAGGGCAGCGGCAGCACCGCCAACCTGGCCAGCAGCAACTACTTCCCTACCCCCAGCGGCTCCATGGTGACCAGCGACGCCCAGATCTTCAACAAGCCCTACTGGCTCCAGAGAGCCCAGGGCCACAACAACGGCATCTGCTGGGGCAACCAGCTGTTCGTGACCGTGGTGGATACCACCCGGAGCACCAACATGTCCCTGTGCGCCGCCATCAGCACCAGCGAGACCACCTACAAGAACACCAACTTCAAGGAGTACCTGAGGCACGGCGAGGAGTACGACCTCCAGTTCATCTTCCAGCTGTGCAAGATCACCCTCACCGCCGACGTGATGACCTACATCCACAGCATGAACAGCACCATCCTGGAGGACTGGAACTTCGGCCTGCAGCCCCCTCCTGGCGGCACCCTGGAGGACACCTACAGATTCGTGACCAGCCAGGCCATCGCATGCCAGAAGCACACCCCTCCCGCCCCTAAGGAGGACCCCCTGAAGAAGTACACCTTCTGGGAAGTGAACCTGAAGGAGAAGTTCAGCGCCGACCTGGACCAGTTCCCTCTGGGCAGAAAGTTCCTGCTGCAGGCCGGCCTGAAGGCCAAGCCTAAGTTCACCCTGGGCAAGAGAAAGGCCACCCCCACCACAAGCAGCACCAGCACCACCGCCAAGCGGAAGAAGCGCAAGCTGTGA

**Human codon-optimized HPV-16 (h)L1 CDS and Physcomitrella-optimized HPV-16 (p)L1 CDS.** Grey marked nucleotides were changed in HPV-16 pL1 through codon usage optimization by the tool physCO (Top et al. 2021). Turquoise marked motifs refer to the Physcomitrella alternative splicing recognition sites. The green marked segment refers to a predicted miRNA binding site identified by the tool psRNATarget (Dai and Zhao 2011; Dai et al. 2018). Light orange colored segments represent the nuclear localization signal. Nucleotide changes are underlined in the HPV-16 pL1 sequence. (hL1, GenBank accession number DQ067889; Maclean et al. 2007).

## **Supplementary Figure S3 - Characterization of CTPs by analysis of subcellular eGFP localization**


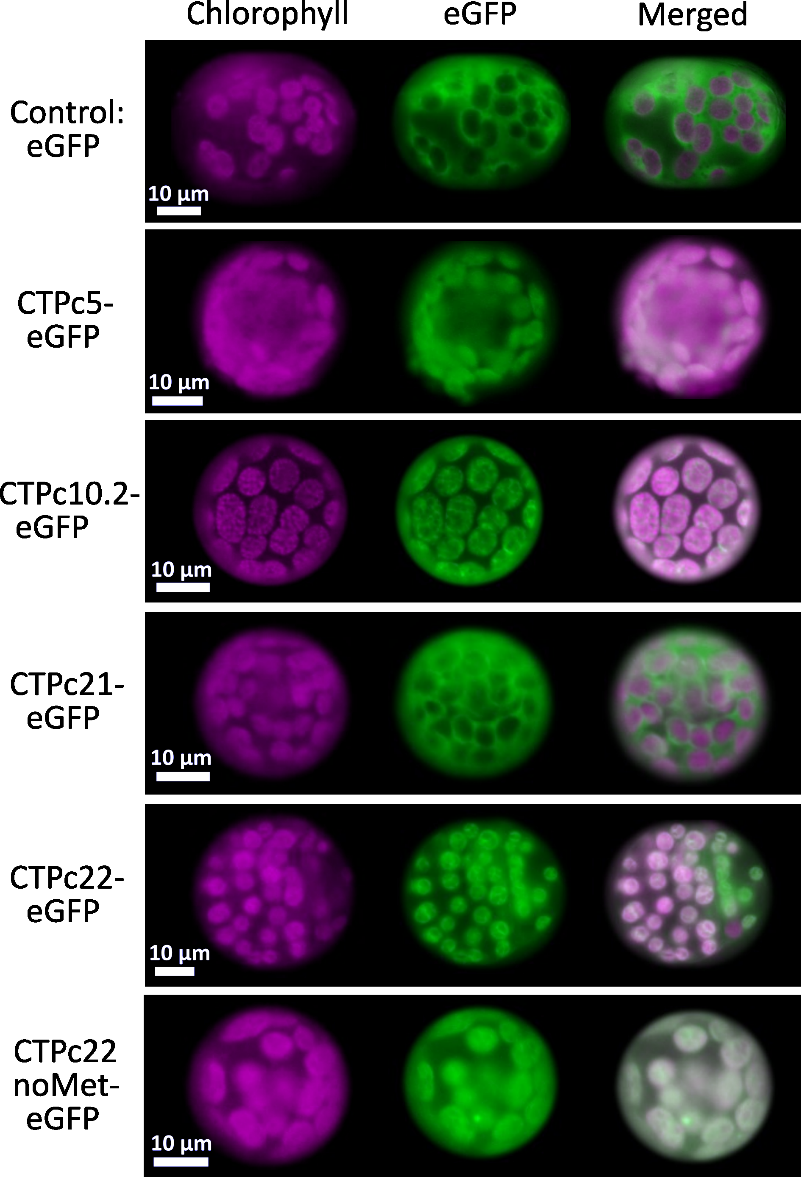


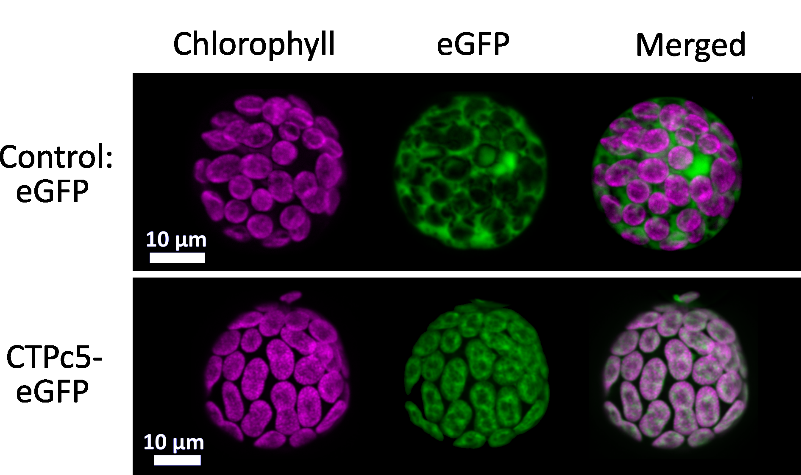


**Microscopy pictures of Physcomitrella protoplasts transformed with CTP-eGFP constructs.** Samples were analysed using a rhodamine and a FITC filter to identify chloroplast (magenta) and eGFP localization, respectively, as well as a merge of the two signals. For sample CTPc21-eGFP no eGFP signal could be observed in chloroplasts. For samples CTPc5-eGFP, CTPc10-eGFP, CTPc22-eGFP and CTPc22noMet-eGFP green fluorescence signal is observed in chloroplasts. eGFP lacking a CTP localized to the cytoplasm and served as a negative control. The experiment was repeated two times. CTPc5 was examined a third time using confocal microscopy. Scale bars represent 10 µm.

## **Supplementary Figure S4 – Analysis of CDS integrity in L1-producing lines**

**
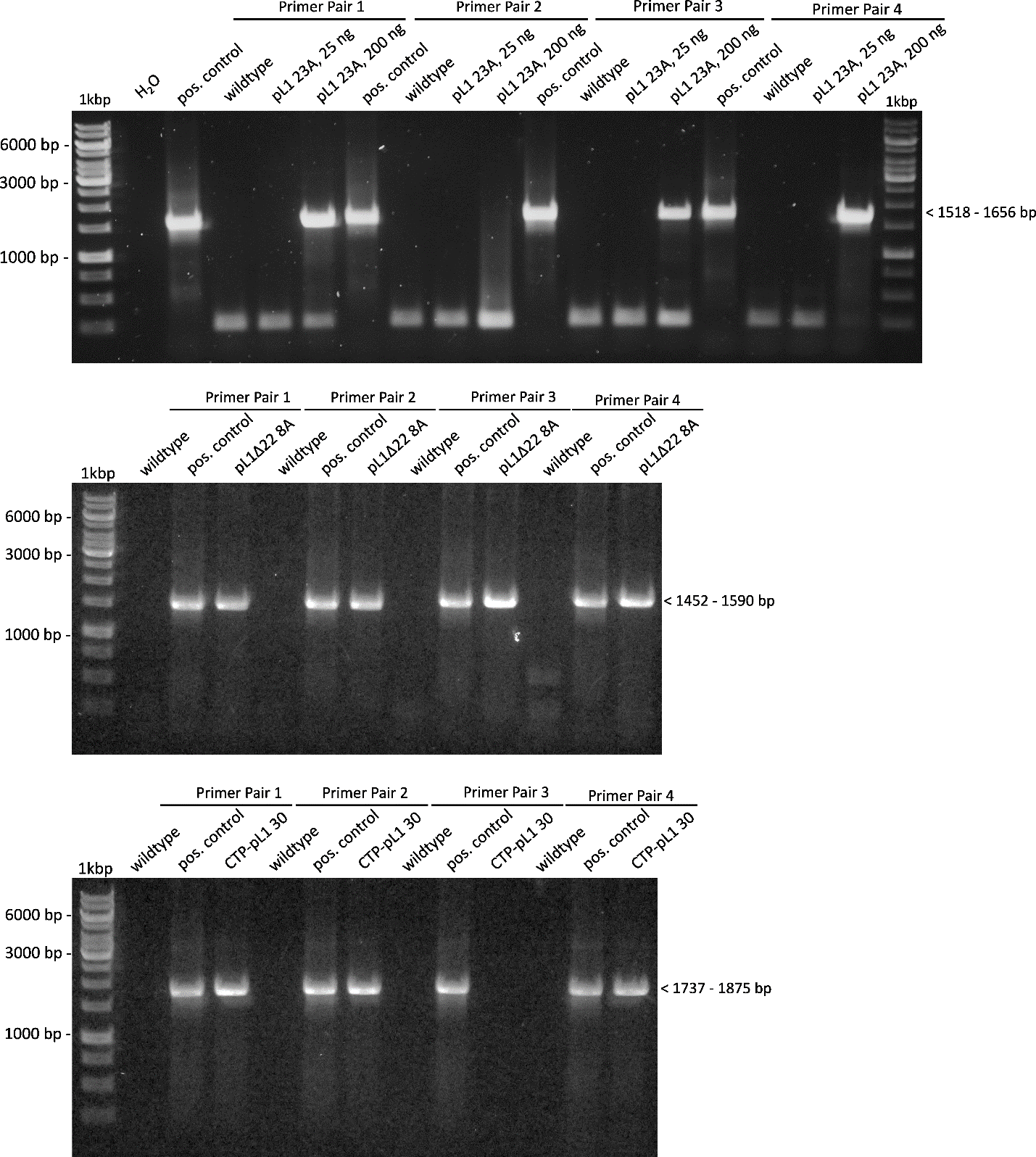
**

**Analysis of rearrangements in the pL1 CDS using RT PCR.** Lines pL1 23A, pL1Δ22 8A and CTP-pL1 30 were checked for DNA or RNA rearrangements by performing PCR on cDNA using four different primer pairs. Primer pairs were selected to amplify the entire cDNA. The forward primer binds at the start codon and is the same in each primer pair (pL1_fwd_1 for lines pL1 23A & pL1Δ22 8A, CTP_pL1_fwd_1 for line CTP-pL1 30). The reverse primers are different in each pair and bind at the 3’ end of the CDS or within the terminator (**Supplementary Table S1**). For line pL1 23A usage of 25 ng or 200 ng cDNA in PCR reactions was compared. Following experiments used 200 ng. Absence of distinct bands of a size smaller than the expected size is indicative for no larger rearrangements on both DNA and RNA level. No amplicon is visible for lines pL1 23A using Primer Pair 2 and CTP-pL1 30 using Primer Pair 3. The constructs used for transformation of Physcomitrella protoplasts (**Supplementary Figure S1 g - i**) served as a positive control.

## **Supplementary Figure S5 – Analysis of the effect of reducing agents on L1 band patterning**


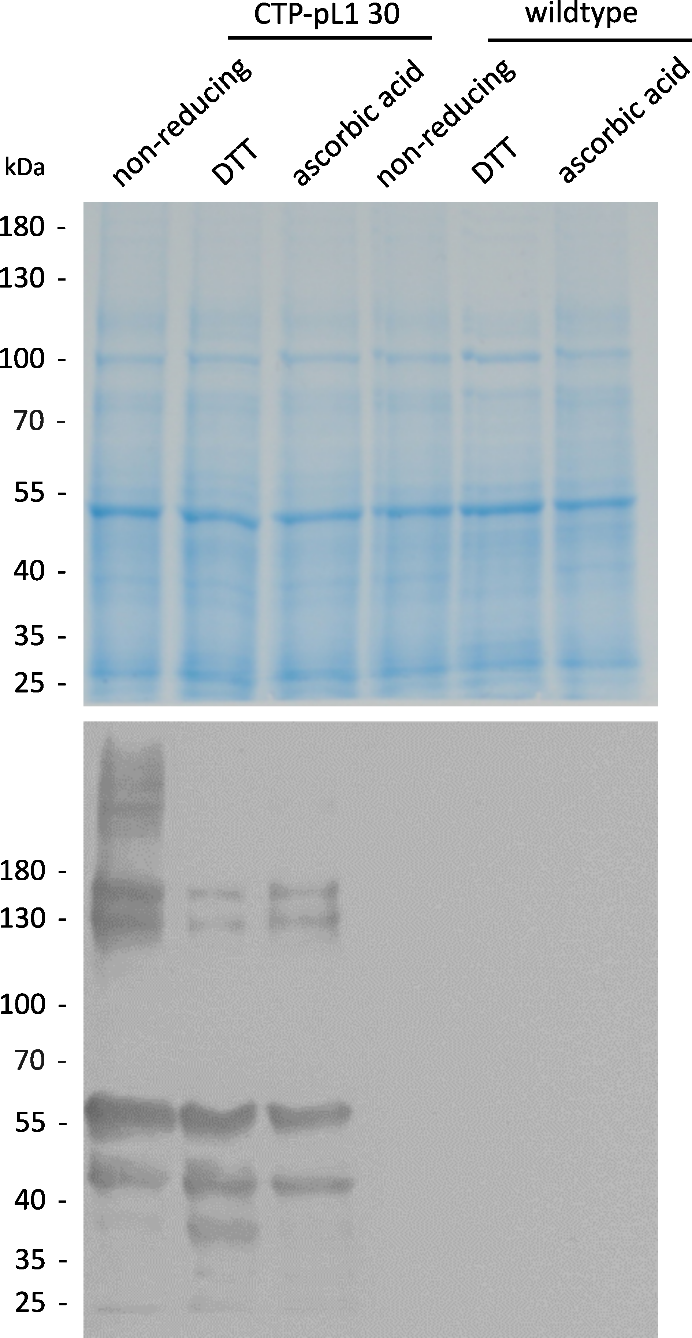


**Analysis of the effect of reducing agents on L1 band patterning in western blots.** Samples of line CTP-pL1 30 and wildtype were extracted without reducing agent, with 50 mM DTT or 40 mM ascorbic acid. The proteins for the western blot were separated on a 7.5% TGS polyacrylamide gel under reducing conditions (5 mM DTT) before being transferred to a membrane and developed using CamVir1 antibody (1:5000).

## **Supplementary Figure S6 – Comparison of biomass accumulation rates**


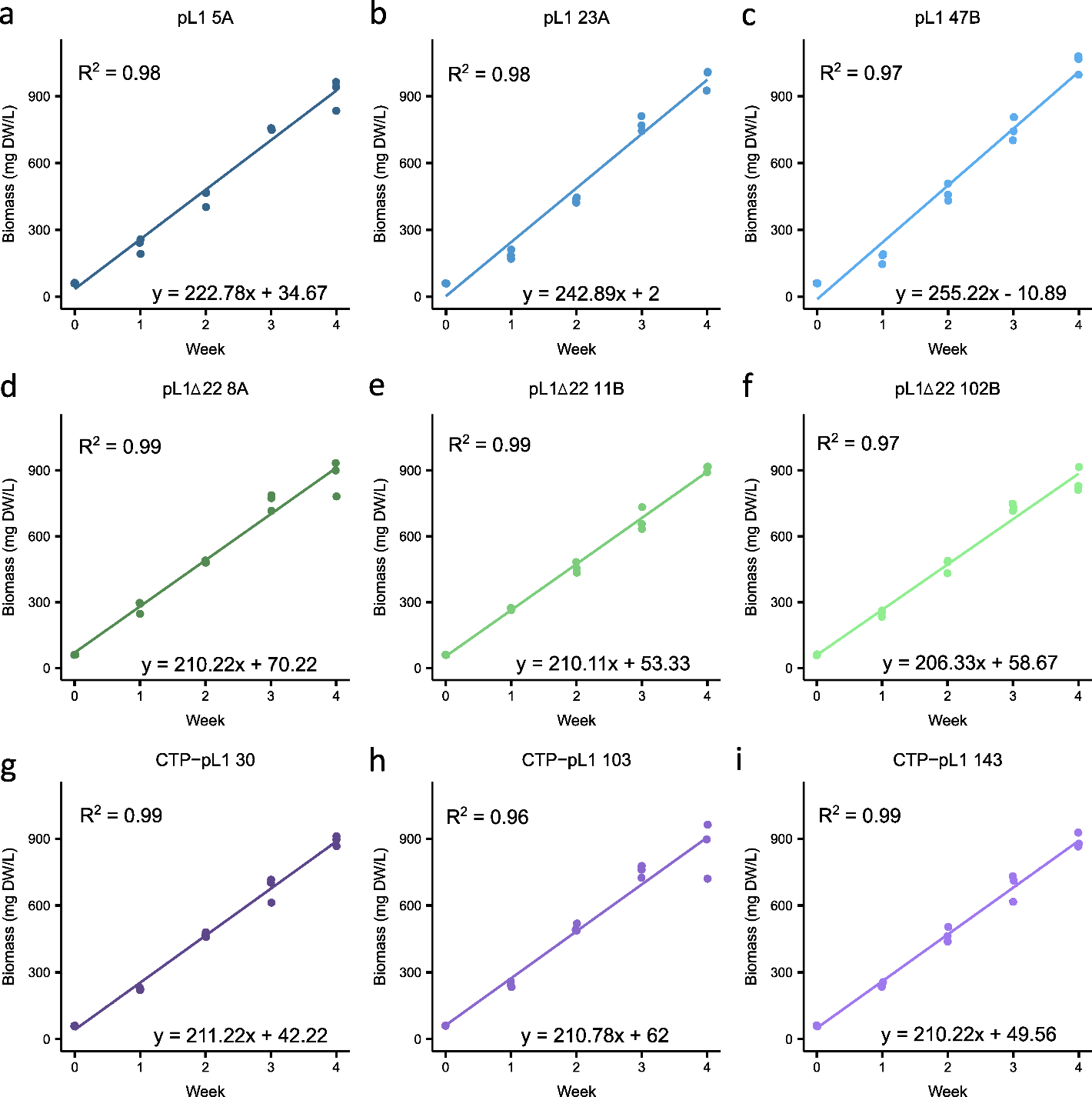


**Analysis of biomass accumulation rates among nine Physcomitrella lines of three different constructs.** Biomass accumulation rates were observed over a course of 4 weeks for lines pL1 5A, pL1 23A, pL1 47B (blue); pL1Δ22 8A, pL1Δ22 11B, pL1Δ22 102B (green) and CTP-pL1 30, CTP-pL1 103, CTP-pL1 143 (purple). The data was plotted using linear regression analysis and all slopes were analyzed using ANOVA and post hoc multiple comparisons test (TukeyHSD). Comparison of the slopes between the constructs of pL1, pL1Δ22 and CTP-pL1 showed pL1 lines accumulated biomass faster than pL1Δ22 (p = <0.0001) and CTP-pL1 (p = 0.0001) lines. Per week n = 3 biological samples were taken.

## **Supplementary Figure S7 – Ammonium sulphate precipitation**


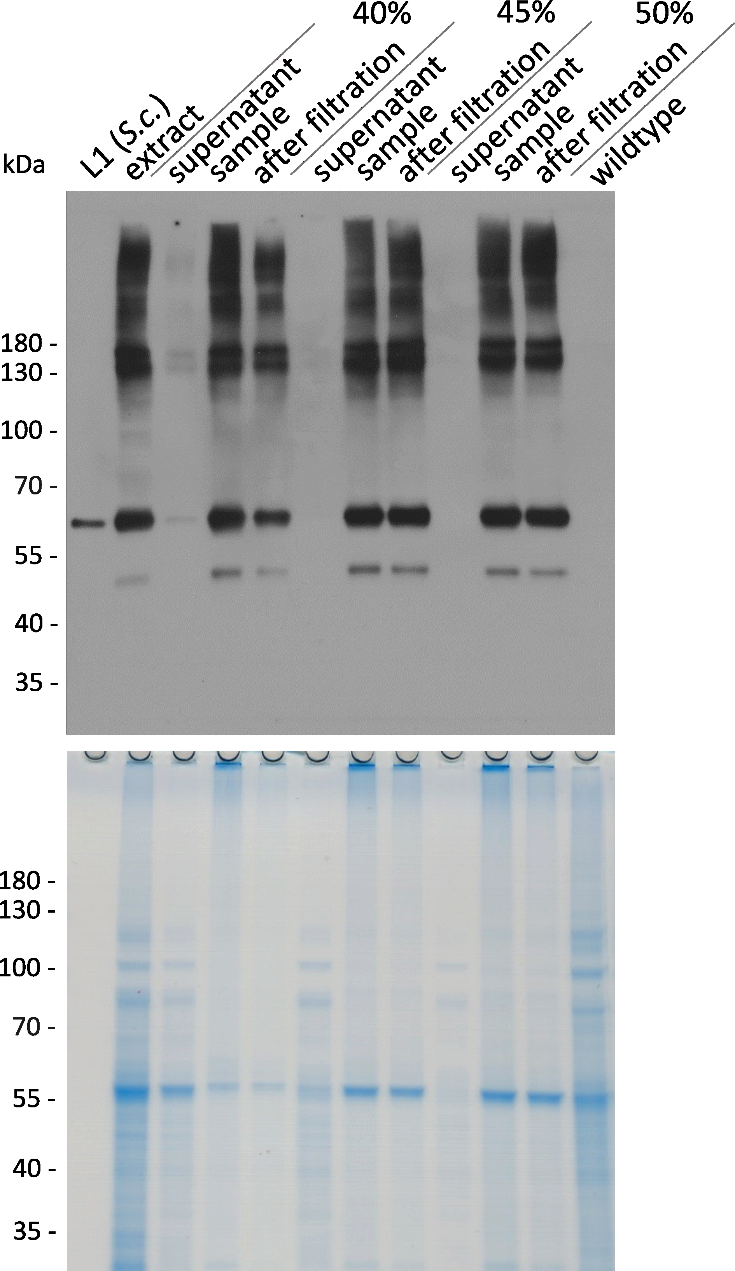


**Ammonium sulphate precipitation of L1 from moss extracts.** Samples from the supernatant and the resuspended and dialyzed pellet before and after filtration were analysed for the presence of L1 content and residual host proteins under reducing conditions *via* western blot and Coomassie-stained gel, respectively. CTP-pL1 30 extracts were precipitated with either 40%, 45%, or 50% (w/v) ammonium sulfate (AMS). After centrifugation, the supernatant and the precipitate were taken up and dialyzed against cation exchange chromatography binding buffer to remove excess AMS. Non-dissolved protein was cleared from the sample using 0.22 µm filters. 45% AMS was found to be sufficient for full precipitation of L1 from crude extracts. 30 ng *S. cerevisiae*-produced L1 served as a positive control and Physcomitrella wildtype as a negative control.

## **Supplementary Figure S8 – Chromatogram of Cation Exchange purification**


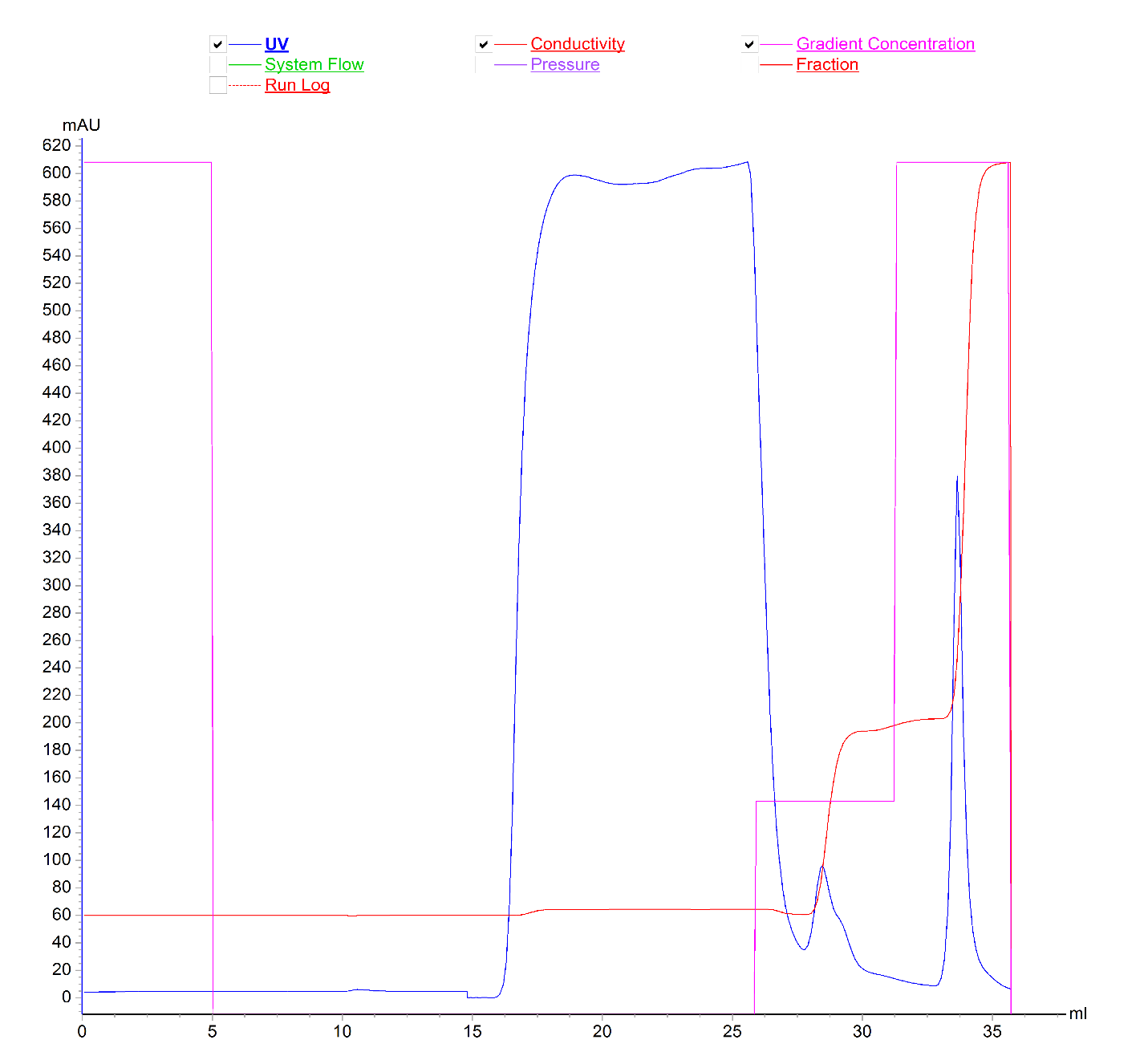


**Chromatogram of HPV-16 L1 purification from moss using cation exchange chromatography.** The chromatogram shows the UV absorbance, indicative for protein concentration, in blue, gradient concentration in pink, and conductivity, indicative for salt concentration, in red. The y axis depicts milli-Absorbance Units and the x axis depicts the volume in mL. The large peak between 16 – 26 mL shows the sample application or flow through, the following small peak at 28 mL the wash fraction and the larger peak at 33 – 35 mL the elution fractions.

## **Supplementary Table S1 – List of primers**

Capital letters refer to the part of the primer binding to the template whereas lower case letters represent overhangs added to the template via PCR. Abbreviations: fwd or fw (forward), rev or rv (reverse), CTP (chloroplast transit peptide), MMEJ (micro homology-mediated end joining), GFP (green fluorescent protein), OH (overhang (lower case letters)).

| Primer name | Sequence (5’🠖3’) | Application |
| --- | --- | --- |
| AA2_3integFP (hpt3'seq) | ACTCGCCGATAGTGGAAACC | Amp of linker-eGFP vector part 1 |
| PAN_PpActin-Pro_Rv | CTGCACAACAACCACCAATATTTAATTTC | Amp of linker-eGFP vector part 1 |
| linker:EGFP_p1840_Fw | GGAGGTGGAGGTGGAGC | Amp of linker-eGFP vector part 2 |
| HPTLUCfwd2 | TTGCCCTCGGACGAGTGC | Amp of linker-eGFP vector part 2 |
| CTP_Pp3c10_Fw | aattaaatattggtggttgttgtgcagATGGCGTTATTGGCGCGC | Amp of CTPc10 |
| CTP_Pp3c10_Rv | gctccacctccacctccATTGGTGACCCTCAAAGGTCCAG | Amp of CTPc10 |
| CTP_Pp3c5_Fw | aattaaatattggtggttgttgtgcagATGGCTACGATGAAGATTGCTGTGG | Amp of CTPc5 |
| CTP_Pp3c5_Rv | gctccacctccacctccAGCTCGCACGGAGCTGTTC | Amp of CTPc5 |
| CTP_Pp3c22_Fw | aattaaatattggtggttgttgtgcagATGCGGGCTCTCTGTGGAG | Amp of CTPc22 & CTPc22_noMet |
| CTP_Pp3c22_Rv | gctccacctccacctccCATAGCGACAATGCACCGTCTTC | Amp of CTPc22 |
| CTP_Pp3c22_noMet_Rv | gctccacctccacctccAGCGACAATGCACCGTCTTCC | Amp of CTPc22_noMet |
| CTP_Pp3c21_Fw | aattaaatattggtggttgttgtgcagATGGCCGCCGTCGGAATG | Amp of CTPc21 |
| CTP_Pp3c21_Rv | gctccacctccacctccGGCACGGACAGCGAACGC | Amp of CTPc21 |
| Act5_seq | TCCACATGGCTACAGCTG | Colony PCR CTP-eGFP constructs |
| EGFP_NoLinker_Rev | CTTGTACAGCTCGTCCATG | Colony PCR CTP-eGFP constructs |
| XhoI-pL1-BamHI_fw | gtgcagctcgagATGTCCCTGTGGCTGCC | Amp of pL1 and pL1Δ22 |
| XhoI-pL1-BamHI _Rv | tctagaggatccTCACAGCTTGCGCTTCTTCC | Amp of pL1 |
| XhoI-pL1delta22-BamHI _Rv | tctagaggatcctcaGCCCAGGGTGAACTTAGGC | Amp of pL1Δ22 |
| pL1_w/oATG_Fw | TCCCTGTGGCTGCCCAG | Amp of CTP-pL1 (no Start codon) |
| pL1_Rv_35St-OH | GACTGGTGATTTTTGCGGACTCTAG | Amp of pL1, Heterosplicing and MMEJ check of pL1 lines |
| CTPc5_Fw_Act5p-OH | tattggtggttgttgtgcagctcgagATGGCTACGATGAAGATTGCTGTG | Amp of CTPc5 |
| CTPc5_Rv_pL1-OH | ctcgctgggcagccacagggaAGCTCGCACGGAGCTGTTC | Amp of CTPc5 |
| Seq_hL1+HSP_Fw | CGTGATGACCTACATCCACAG | Colony PCR pL1 constructs |
| pL1_Rv_35St-OH | GACTGGTGATTTTTGCGGACTCTAG | Colony PCR pL1 constructs |
| pL1_fwd_1 | ATGTCCCTGTGGCTGCCC | Heterosplicing and MMEJ check of pL1, pL1Δ22 lines (primer pair 1-4, forward) |
| pL1_rev_1 | TCACAGCTTGCGCTTCTTCC | Heterosplicing and MMEJ check of pL1, CTP-pL1 lines (primer pair 1, reverse) |
| pL1d22_rev_1 | TCAGCCCAGGGTGAACTTAGG | Heterosplicing and MMEJ check of pL1Δ22 lines (primer pair 1, reverse) |
| CTP-pL1_fwd_1 | ATGGCTACGATGAAGATTGCTGTG | Heterosplicing and MMEJ check of CTP-pL1 lines (primer pair 1-4, forward) |
| pL1_Rv_35St-OH | GACTGGTGATTTTTGCGGACTCTAG | Heterosplicing and MMEJ check of pL1, pL1Δ22, CTP-pL1 lines (primer pair 2, reverse) |
| 101_35S-T_rev | CCCTTATCTGGGAACTACTCACACATTATTCTGGA | Heterosplicing and MMEJ check of pL1, pL1Δ22, CTP-pL1 lines (primer pair 3, reverse) |
| 3342_LdC-HATag-35ST-rev | GGGTTTCGCTCATGTGTTGAGCA | Heterosplicing and MMEJ check of pL1, pL1Δ22, CTP-pL1 lines (primer pair 4, reverse) |
| pL1-fwd | GCTCGAGATGTCCCTGTG | pL1(Δ22)-GFP fusion construct |
| pL1-rev | AGGATCCCAGCTTGCGCTTCTTC | (CTP-)pL1-GFP fusion construct |
| pL1d22-rev | AGGATCCGCCCAGGGTGAACTTAG | pL1Δ22-GFP fusion construct |
| CTPc5-pL1 fwd | GCTCGAGATGGCTACGATGA | CTP-pL1-GFP fusion construct |

## **Supplementary Table S2 – Predicted chloroplast transit peptides for six selected proteins in Physcomitrella. The predictions were performed with TargetP-2.0; Armenteros et al. (2019).**

| Name: CTPc5  Protein: **Pp3c5_1370V3.1**  Predicted chloroplast transit peptide (74 aa):  MATMKIAVAAVAGTPAAAVGTRSAAATETARLCASSAQGLGLSTFVGLRAGSNASRGVTPRVRPLKAENSSVRA |
| --- |
| Name: CTPc10.1  Protein: **Pp3c10_10490V3.1**  Predicted chloroplast transit peptide (63 aa):  MEVLPRAIATSHVVVSSSSAPKESSESGSKNNSQVSLSSVPFLRPASALAASIVSSATPAALA |
| Name: CTPc10.2  Protein: **Pp3c10_2940V3.1**  Predicted chloroplast transit peptide (70 aa):  MALLARGAVAAVAAVGFSNTTSKQGHQNGGSNGVLAFRGNAFCRAVAARTSSVGCQVRVKRGAGPLRVTN |
| Name: CTPc21  Protein: **Pp3c21_9980V3.1**  Predicted chloroplast transit peptide (43 aa):  MAAVGMTMSLKSSTALKGDFLGAALKQTVAAPKAANVAFAVRA |
| Name: CTPc22  Protein: **Pp3c22_5470V3.1**  Predicted chloroplast transit peptide (62 aa):  MRALCGVPSCASLSSHRLASPLLAYRACEFALGSSPGLRLEFVGRAVGLQSRSIGRRCIVAM |
